# Protein-protein binding site identification by enumerating the configurations

**DOI:** 10.1186/1471-2105-13-158

**Published:** 2012-07-06

**Authors:** Fei Guo, Shuai Cheng Li, Lusheng Wang, Daming Zhu

**Affiliations:** 1 School of Computer Science and Technology, Shandong University, Shandong, Jinan 250101, China; 2 Department of Computer Science, City University of Hong Kong, 83 Tat Chee Avenue, Kowloon, Hong Kong

## Abstract

**Background:**

The ability to predict protein-protein binding sites has a wide range of applications, including signal transduction studies, de novo drug design, structure identification and comparison of functional sites. The interface in a complex involves two structurally matched protein subunits, and the binding sites can be predicted by identifying structural matches at protein surfaces.

**Results:**

We propose a method which enumerates “all” the configurations (or poses) between two proteins (3D coordinates of the two subunits in a complex) and evaluates each configuration by the interaction between its components using the Atomic Contact Energy function. The enumeration is achieved efficiently by exploring a set of rigid transformations. Our approach incorporates a surface identification technique and a method for avoiding clashes of two subunits when computing rigid transformations. When the optimal transformations according to the Atomic Contact Energy function are identified, the corresponding binding sites are given as predictions. Our results show that this approach consistently performs better than other methods in binding site identification.

**Conclusions:**

Our method achieved a success rate higher than other methods, with the prediction quality improved in terms of both accuracy and coverage. Moreover, our method is being able to predict the configurations of two binding proteins, where most of other methods predict only the binding sites. The software package is available at http://sites.google.com/site/guofeics/dobi for non-commercial use.

## Background

Most of the existing efforts to identify the binding sites in protein-protein interaction are based on analyzing the differences between interface residues and non-interface residues, often through the use of machine learning or statistical methods. These methods differ in the features analyzed, that is, the sequence and structural or physical attributes. Chung *et al.*[1] used multiple structure alignments of the individual components in known complexes to derive structurally conserved residues. Sequence profile and accessible surface area information are combined with the conservation score to predict protein-protein binding sites by using a Support Vector Machine. Ofran *et al.*[2] employed neural networks to predict binding sites, using the sequence environment, the profile and the structural features as input. The random forest algorithm is used to utilize these features from sequences or 3D structures for the binding site prediction [3,4]. PSIVER [5] uses sequence features for training a Na*ï*ve Bayes classifier to predict binding sites. In PSIVER, conditional probabilities of each sequence feature are estimated using a kernel density estimation method.

Besides the machine learning and statistical approaches, 3D structural algorithms and other methods have also been used to identify binding sites through investigating protein surface structures. ProBiS [6] predicts binding sites by local surface structure alignment. It compares the query protein to 3D protein structures in a database to detect proteins with structurally similar sites on the surfaces. Burgoyne *et al.*[7] analyzed clefts in protein surfaces that are likely to correspond to the binding sites. They ranked them according to sequence conservation and simple measures of physical properties including hydrophobicity, desolvation, electrostatic and van der Waals potentials. Ortuso *et al.*[8] defined most relevant interaction areas in complexes deriving pharmacophore models from 3D structure information. It is based on 3D maps computed by the GRID program on structurally known molecular complexes.

ProMate [9] is based on the idea of interface and non-interface circles. A circle is first created around each residue. Then, features are extracted from these circles. Statistics are performed and histograms are created for each feature. Thereafter, the probability for each circle of a test protein to be an interface is estimated. The interface circles are clustered for each test protein to identify the binding patch.

Bradford *et al.*[10] proposed an approach (PPI-Pred) which uses SVM (Support Vector Machine) on surface patch features to predict binding sites. PPI-Pred generates an interacting patch and a non-interacting patch for each protein. Seven features are extracted for each patch to build an SVM model, which is then used to predict if a given test patch is an interacting patch.

In PINUP [11], an empirical scoring function is presented to predict binding sites. The function is a linear combination of energy score, interface propensity and residue conservation score. A patch is formed by a residue and its spatial neighbors within the protein subunit. PINUP takes the top 5% scoring patches and ranks residues based on their occurrences in these patches. The top 15 ranked residues are predicted as the interface residues.

Li *et al.*[12] proposed another SVM approach (core-SVM). The residues of the proteins are divided into four classes: the interior residues, the core interface residues, the rim interface residues, and the non-interface residues. The core interface and rim interface residues are distinguished by the percentage of their neighboring residues which are interface residues. An SVM is built over eight features extracted from the interface residues, and used to compute the probability of whether a residue is a core interface residue.

Meta-servers have also been constructed to combine the strengths of existing approaches. The program called meta-PPISP [13] combines three individual servers, namely cons-PPISP, ProMate and PINUP; another program called metaPPI [14] combines five prediction methods, namely PPI-Pred, PINUP, PPISP, ProMate, and SPPIDER [15].

Another approach in binding site prediction is to examine the possible structural configurations, or referred to as poses, of protein subunits, that is, how the subunits may dock. Docking methods based on fast Fourier transformation (FFT) [16,17], geometric surface matching [18], as well as intermolecular energy [19-21] have been proposed. Fernández-Recio *et al.*[22] simulated protein docking and analyzed the interaction energy landscapes. Their method uses a global docking method based on multi-start global energy optimization of the ligand. It explores the conformational space around the whole receptor, and uses the rigid-body docking configurations to project the docking energy landscapes onto the surfaces. The low-energy regions are predicted as the binding sites.

In this paper, we propose a method which enumerates the configurations of two binding proteins (that is, the possible positions of the two subunits in a complex), and identify binding sites by evaluating the interaction between the components using the Atomic Contact Energy (ACE) function [23]. We perform *rigid transformation* to enumerate the configurations of two binding proteins. The enumeration is performed in conjunction with a surface identification technique for avoiding clashes between protein subunits when computing rigid transformations. The transformations which result in the minimum score according to the Atomic Contact Energy function are found; the corresponding interacting residues are reported as binding sites. Our method is implemented in a program called DoBi^a^.

We perform experiment to compare DoBi with the existing methods using commonly used measures for assessments. The program outperforms the other methods on these measures. DoBi achieved a success rate higher than all the other methods, improving prediction quality in terms of both accuracy and coverage. In addition, it predicts the configurations of two binding proteins, as opposed to giving only the binding sites.

## Methods

The main idea of our method is to enumerate “all” configurations between two proteins, where a *configuration* refers to the 3D coordinates representing the relative position and orientation of two protein subunits in a complex. We use the Atomic Contact Energy (ACE) function to compute the score for a configuration. The configurations with the lowest score are chosen, and the corresponding interacting residues are predicted as binding sites. We use rigid transformation to enumerate the configurations. The key techniques required here contain (1) an efficient algorithm to enumerate “all” configurations (rigid transformations) and (2) a good energy score.

### Atomic contact energy

Atomic Contact Energy (ACE) is an atomic desolvation energy measure developed in [24]. It is defined over the energy of replacing a protein-atom/water contact, with a protein-atom/protein-atom contact. The ACE score takes into account 18 atom types, hence resulting in 18×18 possible atom pairs. The score for each atom pair has been determined, based on a statistical analysis of atom-pairing frequencies in known proteins. These pre-determined scores are given as log likelihood values in [24], thus allowing the summation of these values. The pre-determined score of effective contact energy between atom type *i* and type *j* is defined as

(1) $$T[i,j]=-\ln\frac{N_{i,j}/C_{i,j}}{\left(N_{i,0}/C_{i,0})\times (N_{j,0}/C_{j,0}\right)}$$

where type 0 corresponds to the solvent. The number of *i*-*j* contact (*N*_*i*,*j*_) and the number of *i*-0 contact (*N*_*j*,0_) are estimates of the actual contact numbers of known complexes. In addition, *C*_*i*,*j*_ and *C*_*i*,0_ are defined as the expected numbers of *ij* contact and *i*-0 contact.

For a given configuration, the ACE score is a summation of each of the atom pairs (one from each subunit) within threshold distance *d*, and *d *= 6*Å* is used in this paper. Denote the sets of atoms from the two subunits as *S*_1_ and *S*_2_, respectively, then the ACE is computed as

(2) $$E_{\text{ACE}}=\underset{s\in S_{1},t\in S_{2},||s-t||\leq d}{\sum }T[s,t]$$

where |*s*−*t*| is the Euclidean distance between *s* and *t*, and *T*[*s*,*t*] is the pre-determined score of the atom pair *s* and *t*.

The ACE score can be considered an estimate of the change in desolvation energy of the two proteins in going from the unbound state to the complex. A lower ACE value implies a lower (and hence more favorable) desolvation free energy.

### Enumeration of the configurations

In this paper, we assume that subunits are rigid. A protein structure consists of a sequence of residues. Each residue consists of a set of atoms. We assume that the atoms in a residue are ordered as a sequence. Hence, the whole protein structure can be represented by a sequence of atoms. In the rest of this subsection, we let *A* and *B* denote two protein structures (subunit), and write *A *= (*a*_1_,*a*_2_,…,*b*_*m*_), and *B *= (*b*_1_,*b*_2_,…,*b*_*n*_), where *a*_*i*_, and *b*_*j *_are atoms of structure *A* and *B*. Without loss of generality, we assume that *n *≥* m*. We also assume that we know the 3D coordinates of each atom in both input proteins. We use *A*[*i*:*j*] to denote the subsequence (*a*_*i*_,…,*a*_*j*_), and refer to a subsequence of atoms as a *structural fragment*.

To enumerate all the configurations, we assume *B* is fixed, and we perform rotations and translations (referred to as rigid transformations, and simply, transformations, in the rest of the paper) on *A*. The method proposed here is modified from the algorithms for structure comparison [25].

**Figure 1:**
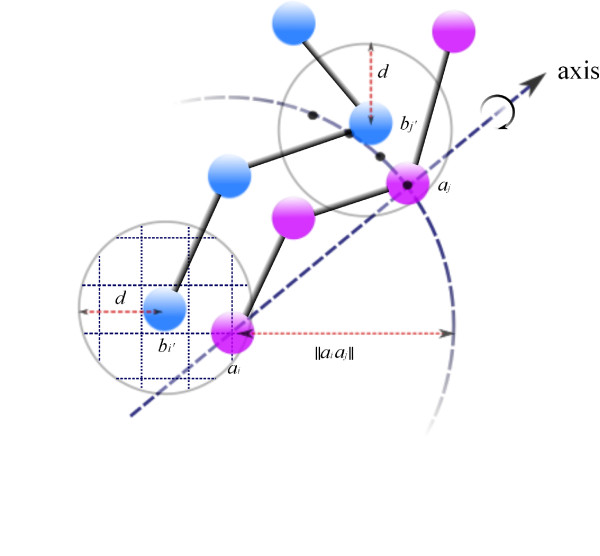
**Steps to obtain a transformation.** (1) put *a*_*i *_at one of the $O({\left(\frac{1}{\varepsilon }\right)}^{3})$ grid points *d*-ball of *b*_*i′*_. (2) put *a*_*j *_at a grid point on the intersection of the sphere centered at *a*_*i *_with radius |*a*_*i*_*a*_*j*_| and *d*-ball of *b*_*j′*_. There are at most $O({\left(\frac{1}{\varepsilon }\right)}^{2})$ grid points on the intersection. (3) use *a*_*i *_and *a*_*j *_as the rotation axis.

Assume that two points *a*_*i *_and *a*_*j*_ of *A* interact with two points *b*_*i′*_ and *b*_*j′*_ of *B*, then we know that ||*a*_*i *_− *b*_*i′*_|| ≤* d* and ||*a*_*j*_ − *b*_*j*_′ || ≤ *d*. To enumerate the configurations, we enumerate the positions for atoms *a*_*i*_ and *a*_*j*_ first, and for each fixed positions of *a*_*i*_ and *a*_*j*_, we rotate *A* about the line formed by *a*_*i *_and *a*_*j*_. Let the *d-ball* of an atom *a* be the ball with radius *d* centered at *a*. We discretize the *d*-ball of *b*_*i′*_ with step size *εd*, where *ε* is a small constant (and we choose *ε *= 0.1 for this paper). Each grid point in the *d*-ball of *b*_*i′*_ is used as a candidate position for atom *a*_*i*_ for the binding. When *a*_*i*_ is fixed at one of the grid points, the possible positions for *a*_*j*_ form a sphere cap, where the sphere is centered at *a*_*i *_with radius |*a*_*i*_−*a*_*j *_|, and the cap is the portion of the spheres enclosed in the *d*-ball of *b*_*j′*_. Again, we discretize the sphere cap with step size *εd*. Each grid point on the sphere cap is a candidate position for *a*_*j*_. This gives us a total of $O({\left(\frac{1}{\varepsilon }\right)}^{5})$ possible positions for the pair of *a*_*i *_and *a*_*j*_. After *a*_*i *_and *a*_*j *_are fixed on their respective grid points, the only degree of freedom to move *A*[*i*,*j*] is to rotate it around the axis through *a*_*i *_and *a*_*j*_. We use a 1° step size; that is, we explore 360 different positions for the remaining atoms through 360 rotations. Figure 1 illustrates the steps to compute a transformation.

The method will work well if we know two interaction pairs (*a*_*i*_,*b*_*i′*_) and (*a*_*j*_,*b*_*j′*_). We can simply enumerate all the atoms pairs as the interaction pair candidate. However, there will be *O*(*n*^4^) such cases, which makes the computer program too slow in practice. This is perhaps one of the reasons that such a method has not been tried. The focus of the following subsection is to identify two pairs (*a*_*i*_,*b*_*i′*_) and (*a*_*j*_,*b*_*j′*_) which are more likely to be interaction pairs.

When enumerating “all” configurations, we also want to make sure that (1) only surface fragments can be candidate binding sites for a configuration and (2) there is no clash between the two proteins in such a configuration. Before presenting the details of the method, we define the *surface atoms* and *clashes of two subunits* first.

### Surface atoms

The interface residues of two proteins are necessarily surface residues. Inspired by the work in LIGSITE_*csc*_[26,27], we propose a method to identify the surface atoms of a protein.

First, we build a 3D grid with step size 1Å around the protein. Then, each grid point is labeled as a *protein* point if it is within distance 2Å of any atom, and labeled as *empty* otherwise. We further subdivide the *protein* grid points into two types: *interior* or *surface*. A *protein* grid point is labeled as *surface* if at least one of its six neighboring grid points is *empty*, otherwise it is labeled as *interior*. With the grid points labeled, we can label the atoms. an atom is labeled as a *surface* atom if it is within distance 1.5Å of a *surface* grid point, otherwise it is labeled as an *interior* atom.

**Figure 2:**
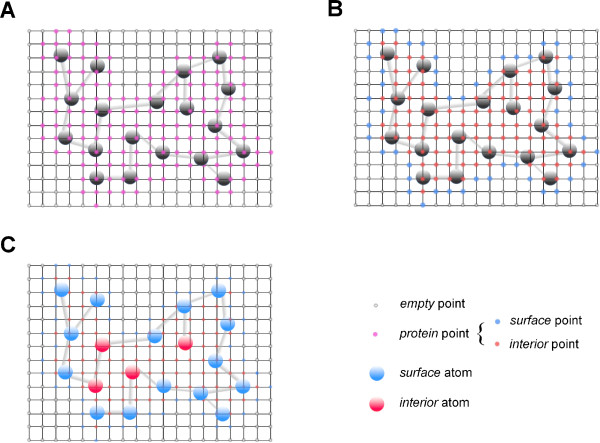
**The surface atoms are indicated in 2D.** (**A**) the grid is created, and grid points are labeled as either *empty* or *protein*; (**B**) the grid points labeled as *protein* are relabeled as *surface* or *interior*; (**C**) an atom is labeled either as *surface* or as *interior*. We use 2D as an illustration.

Figure 2 gives an example in 2D, where a *protein* grid point is labeled as *interior* if it has all four neighbors as *protein* points. In 3D, a *protein* grid point should be labeled as *interior* if all of its six neighbors are labeled as *protein*.

### Clashes of two subunits

A configuration cannot result in two subunits to have clashes. The following method is used to capture if a configuration resulted in clashes. Given a configuration, we build a 3D grid as in the previous subsection. For each of the structures A and B, we mark the grid points as *interior*, *surface*, or *empty*. We use a threshold *θ* to identify whether two subunits clash, by calculating the proportion of interior points for both of them. We say that the two subunits clash if they share more than *θ *× 100*% *of their interior points; that is, if *X* is the number of *interior* grid points which are shared by both proteins, and *X*_*A*_ and *X*_*B *_are the number of *interior* grid points of each subunit, respectively, then we require that *X *≤* θ *×* min*{*X*_*A*_,*X*_*B*_} if the subunits do not clash.

### Finding the two interaction pairs

In the following subsections, we present the details to explore the potential interaction pairs.

#### Identify candidate fragment pairs

We first select fragment pairs that are potential binding sites. As discussed in Section “Enumeration of the configurations”, there are *O*(*n*^4^) possible fragment pairs (*a*_*i*_, *a*_i′_) and (*b*_*j*_, *b*_j′_) for each binding site. To reduce the computational complexity, we adopt a local alignment algorithm to accelerate this selection. This is a raw estimation and we hope that the actual binding sites are not discarded by this process.

We first use a heuristic to quickly discard fragments pairs that are unlikely to bind. The heuristic simplifies the problem, as follows: (1) every atom is within the threshold value required in the ACE computation (that is, we ignore the geometry of the structure); (2) each atom interacts with at most one atom; (3) interacting pairs follows a sequential order. That is, for any two pairs of interacted atoms (*a*_*i*_, *b*_*i′*_) and (*a*_*j*_, *b*_*j′*_), we have either *i *<* i*^*′ *^and *j *<* j*^*′*^, or *i*^*′ *^<* i* and *j*^*′ *^<* j*. With these three simplifications, the standard Smith-Waterman local alignment algorithm [28] can be employed, with the ACE scores used as the penalty (negation of the score) for alignment. We use a penalty of 1 for aligning an atom to a space. Each local aligned segment gives us two fragments, where each atom in the fragment is either aligned to another atom from the partner, or aligned to nothing (i.e., aligned to space).

We present details here. For two sequences *P*_1_and *P*_2_, an alignment of *P*_1_ and *P*_2_ can be obtained by (1) inserting spaces into the two sequences *P*_1 _and *P*_2_ such that the two resulting sequences with inserted spaces *P′*_1_ and *P′*_2_ have the same length and (2) overlap the two resulting sequences *P′*_1_ and *P′*_2_. The score of the alignment is the sum of the scores for all the columns, where each column has a pair of letters (including spaces) and for each pair of letters there is a pre-defined score. A subsequence *α* of *P*_1_ and a subsequence *β *of *P*_2 _can be formed as a local aligned segment such that the score between *α* and *β* is minimum. Here we want to find all (non-overlapping) pairs of subsequences with a score of at most *x*. For our purpose, we set *x *= 0 throughout the paper.

Due to the simplifications, there are many false positive results, and some of the interaction pairs can be filtered. The latter issue can be handled to some extend by raising the threshold. The former issue is tackled by further refinement in the next subsection. In practice, our program outputs 70 to 120 fragment pairs as potential binding sites, which is much smaller than *O*(*n*^4^), where the number of atoms *n* in a protein is from 500 to a few thousands.

Since a binding site is necessarily on the surface of a subunit, we filter out fragments with only very few atoms on the surface. To achieve this, we use a sliding window of length 15 to parse the aligned fragment pair. For each window, if the surface atoms are at least 2/3 (that is, ten atoms) for both fragments, the fragment pair of this window is kept for further processing and this fragment pair is extracted from the alignment. We continue this process on the un-extracted portion of the alignment. If the window does not contain sufficient surface atoms, we continue at the next window. Our choice of 2/3 comes from observations with a docking decoy set from the Dockground [29], where 94% of the binding sites have more than 2/3 of surface atoms.

#### Identify configurations of fragment pairs

From the fragment pairs obtained in the previous step, a second step is used to further filter out fragment pairs of ACE scores below a threshold. Given two structural fragments *A*[*i*,*j*] = (*a*_*i*_,…,*a*_*j*_), and *B*[*i′*,*j′*] = (*bi′*,…,*bj′*), we assume that *a*_*i*_ interacts with *b*_*i′*_, and *a*_*j*_ interacts with *b*_*j′*_. Using the enumeration method described earlier, we enumerate different configurations for *A* and *B* and compute the corresponding ACE score for the atom sets *A*[*i*,*j*] and *B*[*i*^′^,*j*^′^]. We do not consider any configuration which causes *A* and *B* to clash. In this step, a pair of structural fragment which does not give any configuration with an ACE score below a specified threshold is discarded. In this paper, we define the threshold value as 400, since the ACE scores of actual interface in the docking decoy set from Dockground are all less than 400. After this step, it is unlikely for two protein structures which cannot be bound to have an unfiltered fragment pair.

#### Identify the configuration for the two subunits

In the third step, for each pair of protein structures with at least one remaining fragment pair, we enumerate all the potential configurations for the structures. We want to use the begin and end atoms of the identified fragments for our choice of (*a*_*i*_, *b*_*i′*_) and (*a*_*j*_, *b*_*j′*_) in the enumeration, since these are the atoms that are likely to be interacting. Assuming that there are *k* fragment pairs from the same two proteins left after the filtration of the second step, we will have a maximum of 2*k* distinct atom pairs to choose. Thus, there is a total of at most 2k2 combinations to consider for the choice of (*a*_*i*_, *b*_*i′*_) and (*a*_*j*_, *b*_*j′*_).

When the best configuration is obtained, two residues, one from each subunit, are reported as the interface residues if they can be connected with a pair of atoms within distance 4.5Å. In our search for the best configuration, we also require the configurations to be free from clashes.

## Results and discussion

Three commonly used measures are utilized to assess the performance of DoBi. *Accuracy* and *Coverage* are two common measures to assess the quality of the binding sites adopted by a method [11]. The accuracy of the predicted interface is the fraction of correctly predicted residues over the total number of predicted interface residues; the coverage of the predicted interface is the fraction of correctly predicted interface residues over the total number of actual interface residues. *F-score* ( $F=2\times \frac{\text{Accuracy}\times \text{Coverage}}{\text{Accuracy}+\text{Coverage}}$) is a weighted average of the accuracy and coverage, where an F-score reaches its best score at 1 and worst score at 0. Another common measure is *success rate*, which is defined in [9]. A reported result is claimed as a success if at least half of the predicted residues are actual interface residues; that is, the accuracy is no less than 50%. The success rate is the fraction of successful predicted cases in the total number of predicted proteins.

A protein complex may contain several subunits, and multiple binding sites. Each binding site in a protein complex consists of a pair of subunits. Two residues in a pair of subunits are called *interface residues* if any two atoms, one from each residue, interact. By interact, we mean the distance between the two atoms is less than the sum of the van der Waals radius of the two atoms plus 1Å. The number of residues on interface is referred to as the *interface size*.

### Training set

We use the unbound protein structures from Dockground [29] as the training set to calculate the parameters of DoBi. The docking decoys from Dockground were generated by GRAMM-X scan. The GRAMM-X docking scan was used to generate 102 unbound-unbound complexes and 131 unbound-bound complexes. By excluding the proteins used in the comparison, 36 unbound-unbound complexes and 80 unbound-bound complexes can be used to calculate the value of the threshold *θ*. When we set *θ *= 0.17, the overall F-score of DoBi on the training set is 60.5%, which is the best score that DoBi achieves under different threshold values. The details on the training set are shown in Table 1.

**Table 1:** Details of DoBi on the training set

**Complex**	**F**_***r***_^**a**^	**F**_***l***_^**b**^	**Complex**	**F**_***r***_	**F**_***l***_	**Complex**	**F**_***r***_	**F**_***l***_	**Complex**	**F**_***r***_	**F**_***l***_
1a2x(A:B)	45.8	73.7	1jtd(A:B)	59.5	51.2	1r1k(A:D)	24.5	12.0	1z3g(H:A)	77.4	85.7
1a2y(A:C)	77.8	60.9	1jtp(A:L)	62.9	70.0	1rzr(C:T)	60.5	70.3	1z5s(A:B)	52.2	66.7
1aip(A:C)	64.4	59.1	1jwm(A:D)	61.5	58.8	1s3s(F:G)	61.0	66.7	1z92(A:B)	27.0	46.2
1ava(A:C)	74.2	60.3	1k93(A:D)	37.6	33.7	1sgp(E:I)	53.7	58.3	1zlh(A:B)	62.5	64.0
1bnd(A:B)	53.1	57.1	1kkm(A:I)	51.9	58.5	1shw(B:A)	72.7	76.9	1zm2(A:B)	46.9	52.5
1bzq(A:L)	64.9	75.0	1kps(A:B)	68.8	62.5	1sq0(B:A)	50.0	57.1	2a19(B:A)	76.9	72.2
1c9p(A:B)	61.8	54.5	1ktk(E:A)	30.8	61.5	1sq2(L:N)	73.7	78.8	2a41(A:C)	76.4	90.2
1cgj(E:I)	65.3	63.4	1ku6(A:B)	63.0	83.3	1ta3(B:A)	34.6	53.7	2a42(A:B)	79.1	70.2
1cxz(A:B)	54.5	60.0	1l4d(A:B)	81.0	66.7	1te1(A:B)	78.3	83.6	2a5d(B:A)	73.3	84.4
1d4x(A:G)	59.6	72.7	1m27(A:C)	76.2	78.3	1tk5(A:B)	65.6	47.2	2auh(A:B)	60.0	77.3
1df9(A:C)	45.0	58.3	1ma9(A:B)	12.9	60.3	1tu3(A:F)	82.8	76.9	2b12(A:B)	71.0	57.1
1dhk(A:B)	10.8	57.6	1mbx(A:C)	48.9	64.7	1u0n(A:D)	18.2	19.5	2b3t(B:A)	68.9	59.5
1dkf(B:A)	47.8	68.2	1mr1(A:D)	83.7	77.4	1u0s(Y:A)	89.5	90.9	2b5i(B:A)	78.8	62.5
1dp5(A:B)	74.2	86.8	1mzw(A:B)	55.2	72.7	1u7e(A:B)	26.9	62.5	2bh1(A:X)	60.9	57.9
1eai(B:D)	52.2	70.6	1nby(A:C)	50.0	58.3	1uex(A:C)	27.3	50.0	2bkh(A:B)	74.3	67.9
1efu(C:D)	57.1	70.3	1ncb(L:N)	48.6	30.8	1ujw(A:B)	36.1	82.8	2bkk(A:B)	74.3	52.6
1f5q(A:B)	58.2	63.0	1nmu(A:B)	43.9	51.6	1ul1(X:A)	52.6	51.4	2bnq(D:A)	51.9	34.5
1f6a(B:A)	28.6	47.6	1npe(A:B)	43.1	68.1	1uuz(A:D)	58.8	57.9	2c1m(A:B)	40.4	66.7
1f7z(A:I)	72.7	89.7	1nu9(A:C)	56.7	56.8	1uzx(A:B)	71.0	68.7	2c5d(A:C)	54.2	69.4
1ffg(A:B)	73.3	62.1	1oiu(A:B)	70.8	76.2	1v5i(A:B)	3.8	87.2	2gy7(B:A)	63.2	73.2
1fm9(D:A)	82.6	89.4	1omw(A:B)	75.8	63.4	1v7p(A:C)	50.0	41.4	2hdi(A:B)	9.1	57.1
1fns(L:A)	50.0	28.6	1p3q(R:V)	66.7	80.0	1w98(A:B)	50.7	62.3	2iw5(A:B)	66.1	72.5
1g20(A:E)	45.8	40.8	1p7q(A:D)	63.6	61.5	1wpx(A:B)	58.3	55.2	2j0m(A:B)	81.3	64.3
1g9m(G:L)	38.1	28.6	1p9m(C:B)	85.7	70.6	1wr6(A:E)	89.7	93.3	2jb0(B:A)	66.7	63.2
1h0d(A:C)	16.7	30.0	1pkq(A:E)	27.3	9.1	1wrd(A:B)	56.2	69.2	2omz(A:B)	60.2	71.0
1h59(A:B)	91.7	81.5	1ppf(E:I)	85.1	83.9	1x86(A:B)	52.6	60.4	2p8w(T:S)	56.6	90.3
1i8l(A:C)	83.9	71.4	1qav(B:A)	81.3	78.0	1xdt(T:R)	48.4	90.2	2pav(A:P)	72.0	73.1
1iar(B:A)	76.5	51.6	1qbk(B:C)	41.9	38.6	1xx9(C:A)	54.5	40.0	3bp5(B:A)	70.0	72.0
1jl4(A:D)	40.0	44.4	1qo0(B:A)	31.6	33.3	1yi5(A:F)	83.9	76.5	3ygs(C:P)	64.5	58.1

^a^F_*r *_(%) is the F-score of our method on the receptor proteins.

^b^F_*l *_(%) is the F-score of our method on the ligand proteins.

### Comparison to the existing methods

We divide our comparisons into four separate groups, where in each group we compare a different set of methods. The reason that we cannot compare all the methods with the same data set is due to the unavailability of some methods, in which case the only comparison possible is with the results in the respective publications.

#### Comparison to Fernández-Recio *e*t al.’s method

DoBi is compared to the method introduced by Fernández-Recio *et al.* in [22], using the test data therein, which consists of 43 complexes. The results are reported in Table 2. The overall accuracy and coverage for DoBi are 44.3% and 70.5%. Fernández-Recio *et al. *’s method achieved the overall accuracy and coverage of 39.3% and 72.7%, respectively. The success rate for DoBi is 39.6%, improving over the success rate of 37.2% reported by Fernández-Recio *et al.*. The F-score is 0.54 for DoBi, and 0.51 for Fernández-Recio *et al.*’s method.

**Table 2:** **Comparison of DoBi and Fernández-Recio***et al.***’s method**

	**DoBi**						**Fernández-Recio *et al.*’s**					
	**Suc**^**a**^	**Acc**^**b**^	**Cov**^**c**^	**F**^**f**^	***M***^**d**^	***V***^**e**^	**Suc**	**Acc**	**Cov**	**F**	***M***	***V***
Overall	39.6	44.3	70.5	0.54	37.5	29.0	37.2	39.3	72.7	0.51	46.3	40.0

^a^Suc (%) is the success rate of the corresponding method on the data set.

^b^Acc (%) is the average accuracy of the corresponding method on the data set.

^c^Cov (%) is the average coverage of the corresponding method on the data set.

^d^*M* is the average of the sizes predicted by the corresponding method on the data set.

^e^*V* is the standard deviation of the sizes predicted by the corresponding method on the data set.

^f^F is the F-score of the corresponding method on the data set.

The average predicted sizes for DoBi and Fernández-Recio *et al.*’s method are 37.5 residues and 46.3 residues respectively, while the average actual size is 21.1 residues. The standard deviation of the sizes predicted by DoBi is 29.0, while that of the sizes predicted by Fernández-Recio *et al.*’s method is 40.0.

Table 3 displays the detailed results for all unbound structures of 43 complexes. Each row corresponds to a pair of proteins. We can observe from the table that the binding sites are identified accurately for the complexes 2sni(E:I), 2sic(E:I), 1ay7(A:B) and 1wq1(G:R).

**Table 3:** **Detailed Results of DoBi and Fernández-Recio *et al.*’s method**

	**Receptor**						**Ligand**					
**Complex**			**DoBi**		**Fernández-Recio**^**e**^					**DoBi**		**Fernández-Recio**^**e**^	
	**PDB**^**a**^	***Int***_**n**_^**b**^	***Acc***^**c**^	***Cov***^**d**^	***Acc***	***Cov***	**PDB**	***Int***_**n**_	***Acc***	***Cov***	***Acc***	***Cov***	
1ca0(B:D)	5cha	24	46.2	50.0	50.6	81.0	1aap	14	26.1	42.9	35.6	57.0
1cbw(B:D)	5cha	26	58.6	65.4	65.7	92.0	1bpi	14	77.8	100	33.7	64.0
1acb(E:I)	5cha	24	14.5	66.7	55.0	77.0	1egl	13	20.4	84.6	21.6	41.0
1cho(F:I)	5cha	25	36.9	96.0	63.6	89.0	1omu	13	35.3	92.3	48.1	77.0
1cgi(E:I)	1chg	24	26.3	45.5	70.8	92.0	1hpt	19	48.5	84.2	58.3	70.0
2kai(A:I)	2pka	33	53.8	58.3	41.5	54.0	1bpi	19	68.8	84.6	35.9	79.0
2sni(E:I)	2st1	28	61.1	78.6	35.8	93.0	2ci2	15	70.6	80.0	37.9	53.0
2sic(E:I)	2st1	30	73.5	83.3	29.6	83.0	3ssi	12	62.5	83.3	18.4	46.0
1cse(E:I)	1sbc	30	42.6	96.7	33.1	96.0	1egl	12	26.3	83.3	22.8	41.0
2tec(E:I)	1thm	28	38.0	67.9	34.2	82.0	1egl	13	31.0	69.2	30.0	45.0
1taw(A:B)	5ptp	26	42.1	30.8	51.9	83.0	1aap	13	47.1	61.5	34.4	62.0
2ptc(E:I)	5ptp	24	33.3	50.0	52.4	89.0	1bpi	14	56.5	92.9	18.0	36.0
3tgi(E:I)	1ane	25	51.9	56.0	16.1	29.0	1bpi	14	58.8	71.4	30.5	64.0
1brc(E:I)	1bra	24	30.0	25.0	44.4	80.0	1aap	11	62.5	90.9	36.5	62.0
1fss(A:B)	2ace	25	32.7	64.0	23.8	100	1fsc	19	65.4	89.5	69.2	83.0
1bvn(P:T)	1pif	31	29.2	22.6	45.0	90.0	2ait	20	42.1	80.0	61.4	86.0
1bgs(B:F)	1a2p	18	23.1	66.7	73.1	95.0	1a19	16	34.1	93.8	72.3	94.0
1ay7(A:B)	1rge	15	81.3	86.7	71.4	100	1a19	15	84.6	73.3	52.2	94.0
1ugh(E:I)	1akz	24	63.6	87.5	44.1	97.0	2ugi	25	57.1	64.0	83.3	75.0
2pcb(A:B)	1ccp	10	23.5	40.0	24.2	92.0	1hrc	9	22.2	44.4	29.2	73.0
2pcf(B:A)	1ctm	21	57.7	71.4	57.5	92.0	1ag6	24	56.7	70.8	66.4	73.0
1mlc(B:E)	1mlb	14	65.0	92.9	31.3	100	1lza	10	43.5	100	9.1	29.0
1vfb(A:C)	1vfa	8	44.4	100	52.6	100	1lza	8	43.8	87.5	26.8	83.0
1ewy(A:C)	1que	15	20.8	26.3	52.6	100	1fxa	15	37.5	52.9	56.7	68.0
1eer(B:A)	1ern	23	13.8	65.2	35.0	91.0	1buy	22	21.9	95.5	53.6	75.0
1kkl(A:H)	1jb1	13	31.3	76.9	3.5	11.0	1sph	12	32.4	100	67.5	81.0
1ken(A:C)	2viu	56	92.6	44.6	30.3	97.0	1ken	64	71.7	51.6	29.4	100
1kxv(A:C)	1pif	19	15.0	63.2	3.7	10.0	1kxv	21	27.0	81.0	43.7	83.0
1kxt(A:B)	1pif	17	17.9	41.2	14.1	55.0	1kxt	20	30.8	40.0	53.3	96.0
1kxq(A:H)	1pif	30	42.5	56.7	52.6	100	1kxq	25	54.5	72.0	56.5	96.0
1l0x(A:B)	1bec	19	42.9	40.0	0	0	1b1z	17	27.8	41.7	16.1	100
1avw(A:B)	2ptn	31	31.3	48.4	58.8	100	1ba7	15	44.1	100	36.2	94.0
1dfj(I:E)	2bnh	33	52.9	54.5	49.4	89.0	7rsa	29	47.1	55.2	66.7	80.0
1tgs(Z:I)	2ptn	30	30.4	70.0	62.0	93.0	1hpt	18	43.8	77.8	68.3	82.0
1ahw(A:B)	1fgn	43	23.0	39.5	15.6	89.0	1boy	45	28.3	62.2	0	0
1dqj(A:C)	1dqq	11	50.0	81.8	20.0	100	3lzt	11	50.0	81.8	14.4	39.0
1wej(H:F)	1qbl	7	38.9	100	24.4	100	1hrc	8	40.0	100	18.3	44.0
1avz(B:C)	1avv	16	58.8	62.5	16.2	42.0	1shf	13	42.3	84.6	54.1	92.0
1wq1(G:R)	1wer	33	70.6	72.7	11.4	33.0	5p21	26	77.8	80.8	40.8	53.0
2mta(L:A)	2bbk	13	57.9	84.6	30.0	93.0	1aan	11	64.7	100	58.8	100
1bth(H:P)	2hnt	30	15.2	16.7	27.7	61.0	6pti	17	94.1	94.1	32.5	39.0
1fin(A:B)	1hcl	46	35.5	47.8	28.3	68.0	1vin	35	32.8	60.0	66.7	100
1fq1(B:A)	1b39	16	63.2	75.0	8.2	32.0	1fpz	16	63.2	75.0	0	0

^a^PDB is the unbound structure of the receptor or ligand in the complex.

^b^*Int*_*n*_ is the number of residues on the actual interface in the complex.

^c^*Acc* (%) is the accuracy of the corresponding method on the data set.

^d^*Cov* (%) is the coverage of the corresponding method on the data set.

^e^The values for this method are from literature [22].

#### Comparison to metaPPI, meta-PPISP and PPI-Pred

In this group of our comparisons, the test set in [14] is used. It consists of 41 complexes from the benchmark v2.0 [30] and 27 targets from the CAPRI experiment [31]. The 41 complexes are divided into two categories, enzyme-inhibitor (EI) and *others*. We compare our method to metaPPI, meta-PPISP and PPI-Pred with this group of data. The overall accuracy and coverage of each prediction method are shown in Table 4. DoBi has an F-score of 0.55, where in contrast, metaPPI, meta-PPISP and PPI-Pred have the F-scores 0.35, 0.43 and 0.32 respectively. DoBi has a success rate of 53.7%, as well as overall accuracy and coverage of 50.0% and 60.0% respectively.

**Table 4:** Comparisons of DoBi, metaPPI, meta-PPISP and PPI-Pred

	**DoBi**						**metaPPI**						**meta-PPISP**						**PPI-Pred**																								
**Type**	**Suc**^**a**^	**Acc**^**b**^	**Cov**^**c**^	**F**^**g**^	***M***^**e**^	***V***^**f**^	**Suc**	**Acc**	**Cov**	**F**	***M***	***V***	**Suc**	**Acc**	**Cov**	**F**	***M***	***V***	**Suc**	**Acc**	**Cov**	**F**	***M***	***V***																			
E-I^d^	67.6	56.7	61.9	0.59	23.0	7.6	70.5	61.1	36.5	0.45	12.9	10.4	55.8	56.4	54.7	0.55	24.1	13.5	47.1	39.5	37.9	0.38	23.7	15.1																			
others	47.9	46.4	63.3	0.53	29.5	19.8	43.8	40.7	22.2	0.28	8.0	10.1	35.6	38.5	25.7	0.30	11.8	12.6	22.9	29.3	31.3	0.30	19.0	14.7																			
CAPRI	50.0	48.9	55.8	0.52	25.7	12.3	50.0	46.7	24.3	0.32	15.7	12.8	26.0	27.9	30.8	0.29	19.6	13.8	28.6	25.7	29.5	0.27	28.2	19.2																			
Overall	53.7	50.0	60.0	0.55	26.4	13.8	52.9	48.2	26.6	0.35	12.3	11.2	36.8	38.8	35.0	0.43	18.0	13.3	31.2	30.4	32.2	0.32	23.8	16.6																			

^a^Suc (%) is the success rate of the corresponding method on the data set.

^b^Acc (%) is the average accuracy of the corresponding method on the data set.

^c^Cov (%) is the average coverage of the corresponding method on the data set.

^d^E-I is the type of enzyme-inhibitor.

^e^*M* is the average of the sizes predicted by the corresponding method on the data set.

^f^*V* is the standard deviation of the sizes predicted by the corresponding method on the data set.

^g^F is the F-score of the corresponding method on the data set.

The detailed results on all the unbound structures of the 41 complexes are displayed in Table 5. The detailed results on 27 CAPRI targets are displayed in Table 6. Each row displays the results of the methods tested on the two corresponding binding partners.

**Table 5:** Detailed Results of DoBi, metaPPI, meta-PPISP and PPI-Pred on 41 complexes

**Complex**	**Protein 1**									**Protein 2**										
	**PDB**^**a**^	***Int***_**n**_^**b**^	**DoBi**		**metaPPI**^**f**^		**meta-PPISP**^**f**^		**PPI-Pred**^**g**^		**PDB**	***Int***_**n**_	**DoBi**		**metaPPI**		**meta-PPISP**		**PPI-Pred**	
			***Acc***^**c**^	***Cov***^**d**^	***Acc***	***Cov***	***Acc***	***Cov***	***Acc***	***Cov***			***Acc***	***Cov***	***Acc***	***Cov***	***Acc***	***Cov***	***Acc***	***Cov***
E-I^e^																				
1acb(E:I)^h^	2cgaB	24	33.3	20.8	87.5	56.0	60.7	68.0	76.0	79.2	1egl_	13	63.2	92.3	66.7	58.8	100	53.6	90.0	69.2
1ay7(A:B)	1rghB	15	75.0	100	27.3	17.6	53.8	33.3	0	0	1a19B	15	60.0	80.0	72.7	53.3	92.9	81.2	0	0
1cgi(E:I)	2cgaB	33	64.3	27.3	100	55.2	56.0	48.2	96.2	75.8	1hpt_	19	93.3	73.7	100	36.8	89.5	77.3	100	63.2
1d6r(A:I)	2tgt_	27	43.8	25.9	54.5	28.6	53.6	71.4	73.9	63.0	1k9bA	13	66.7	92.3	44.4	53.3	35.7	15.2	22.2	15.4
1dfj(E:I)	9rsaB	29	41.0	55.2	64.3	26.5	57.7	48.4	55.0	37.9	2bnh_	33	43.5	60.6	81.3	31.0	32.4	91.7	21.3	30.3
1e6e(A:B)	1e1nA	20	42.3	55.0	0	0	26.9	43.8	14.9	55.0	1cjeD	23	65.2	65.2	93.3	50.0	79.2	73.1	15.4	17.4
1eaw(A:B)	1eaxA	22	21.1	18.2	100	48.0	46.8	60.0	66.7	72.7	9pti_	14	52.6	71.4	100	42.9	95.0	79.2	8.3	7.1
1ewy(A:C)	1gjrA	19	57.1	84.2	9.1	5.3	5.6	8.3	16.7	52.6	1czpA	17	51.6	94.1	57.1	42.1	63.2	63.2	50.0	41.2
1f34(A:B)	4pep_	25	44.8	52.0	30.8	12.5	30.3	52.6	47.5	76.0	1f32A	24	57.9	45.8	72.7	24.2	55.2	69.6	70.4	79.2
1mah(A:F)	1j06B	27	35.9	51.9	16.7	3.4	28.0	63.6	36.6	96.3	1fsc_	21	86.4	90.5	15.8	15.0	33.3	21.9	33.3	28.6
1ppe(E:I)	1btp_	27	64.9	88.9	64.3	42.9	40.9	42.8	0	0	1lu0A	14	63.2	85.7	92.3	75.0	100	56.0	90.0	64.3
1tmq(A:B)	1jae_	28	62.2	82.1	75.0	40.0	36.0	30.0	63.4	92.9	1b1uA	26	57.1	76.9	93.3	56.0	70.4	76.0	0	0
1udi(E:I)	1udh_	26	52.2	46.2	63.6	25.9	48.0	66.7	72.0	69.2	2ugiB	26	94.4	65.4	92.9	56.5	72.7	80.0	85.7	46.2
2pcc(A:B)	1ccp_	13	20.0	23.1	53.8	50.0	26.7	33.3	0	0	1ycc_	14	26.3	35.7	42.9	35.3	37.5	33.3	13.3	14.3
2sic(E:I)	1sup_	26	50.0	46.2	72.7	38.1	81.8	60.0	62.5	76.9	3ssi_	12	84.6	91.7	0	0	100	72.2	0	0
2sni(E:I)	1ubnA	27	66.7	59.3	60.0	33.3	60.0	83.0	66.7	81.5	2ci2I	15	42.9	40.0	57.1	57.1	0	0	76.9	66.7
7cei(A:B)	1unkD	20	76.9	50.0	75.0	35.3	47.4	60.0	75.0	45.0	1m08B	16	64.3	56.3	40.0	37.5	0	0	13.8	25.0
others																				
1ak4(A:D)	2cpl_	17	42.9	35.3	50.0	31.3	33.3	18.8	59.1	76.5	1e6jP	9	30.4	77.8	0	0	0	0	0	0
1atn(A:D)	1ijjB	17	5.3	5.9	0	0	20.7	37.5	0	0	3dni_	24	40.0	33.3	0	0	0	0	66.7	66.7
1b6c(A:B)	1d6oA	20	54.3	95.0	83.3	55.6	40.0	11.1	93.3	70.0	1iasA	20	44.0	55.0	54.5	25.0	31.6	25.0	0	0
1buh(A:B)	1hcl_	16	68.4	81.3	0	0	6.3	11.8	0	0	1dksA	18	75.0	83.3	58.3	38.9	36.4	22.2	100	66.7
1e96(A:B)	1mh1_	14	66.7	85.7	38.5	25.0	46.2	60.0	10.0	14.3	1hh8A	12	73.3	91.7	41.7	35.7	45.5	35.7	0	0
1fq1(A:B)	1fpzF	16	63.2	75.0	0	0	0	0	0	0	1b39A	16	63.2	75.0	0	0	30.0	23.1	17.1	37.5
1fqj(A:B)	1tndC	21	20.7	81.0	70.6	42.9	32.3	35.7	28.6	38.1	1fqiA	24	18.9	58.3	90.9	47.6	42.9	14.3	78.9	62.5
1gcq(B:C)	1griB	14	35.3	42.9	70.0	63.6	38.9	63.6	22.2	14.3	1gcpB	18	78.9	83.3	60.0	40.0	100	33.3	33.3	16.7
1ghq(A:B)	1c3d_	10	41.7	100	0	0	42.9	37.5	0	0	1ly2A	9	47.4	100	0	0	42.9	66.7	8.7	22.2
1grn(A:B)	1a4rA	17	54.2	76.5	33.3	15.0	40.0	40.0	50.0	58.8	1rgp_	22	50.0	54.5	16.7	4.5	100	13.6	78.9	68.2
1h1v(A:G)	1ijjB	24	28.6	41.7	46.2	13.0	35.3	26.1	38.8	76.0	1d0nB	25	43.8	56.0	0	0	40.0	4.9	4.7	12.0
1he1(C:A)	1mh1_	16	48.0	75.0	66.7	30.8	50.0	42.3	0	0	1he9A	21	40.9	42.9	76.5	46.4	33.3	7.1	0	0
1he8(B:A)	821P_	13	20.6	100	0	0	43.8	33.3	26.7	61.5	1e8zA	15	11.1	53.3	42.9	16.7	5.9	5.6	0.6	6.7
1i2m(A:B)	1qg4A	24	14.3	33.3	42.9	21.4	43.8	50.0	15.0	12.5	1a12A	32	15.1	43.8	0	0	50.0	5.1	48.0	75.0
1ibr(A:B)	1qg4A	35	43.2	45.7	73.3	22.0	55.0	22.0	14.3	8.6	1f59A	42	38.9	33.3	7.1	1.8	0	0	10.3	16.7
1kac(A:B)	1nobF	15	68.4	86.7	0	0	15.4	21.1	0	0	1f5wB	21	83.3	95.2	60.0	28.6	71.4	23.8	35.3	28.6
1ktz(A:B)	1tgk_	9	26.7	44.4	45.5	62.5	13.3	25.0	50.0	88.9	1m9zA	12	57.1	100	66.7	80.0	60.0	60.0	33.3	50.0
1kxp(A:D)	1ijjB	34	13.6	8.8	81.3	30.2	45.5	23.3	4.3	5.9	1kw2B	41	32.0	19.5	0	0	75.0	13.0	48.9	56.1
1kxq(H:A)	1kxqH	25	12.1	16.0	91.7	30.6	78.6	30.6	18.2	8.0	1ppi_	30	22.7	16.7	41.7	17.9	20.0	3.6	47.8	73.3
1m10(A:B)	1auq_	24	57.1	50.0	58.3	24.1	65.0	44.8	50.0	45.8	1mozB	29	68.0	58.6	0	0	31.6	18.2	0	0
1qa9(A:B)	1hnf_	16	76.2	100	0	0	27.3	17.6	10.0	12.5	1cczA	16	82.4	87.5	6.7	5.3	22.2	10.5	28.6	25.0
1sbb(A:B)	1bec_	13	54.2	100	0	0	17.6	17.6	0	0	1se4_	11	50.0	100	0	0	50.0	12.5	10.0	27.3
1wq1(R:G)	6q21D	26	61.5	61.5	66.7	32.3	41.7	32.2	76.2	61.5	1wer_	33	62.5	45.5	100	26.5	36.4	11.8	70.0	63.6
2btf(A:P)	1ijjB	26	63.3	73.1	53.3	32.0	25.0	12.0	22.0	42.3	1pne_	23	56.0	60.9	0	0	70.0	28.0	0	0

^a^PDB is the unbound structure of the two proteins in complex.

^b^*Int*_*n*_ is the number of residues on actual interface in complex.

^c^*Acc* (%) is the accuracy of the corresponding method on the data set.

^d^*Cov* (%) is the coverage of the corresponding method on the data set.

^e^E-I is the type of enzyme-inhibitor.

^f^The values for metaPPI and meta-PPISP are from literatures [14].

^g^The results for PPI-Pred are calculated by using the same definition of actual interface with DoBi.

^h^The binding sites between chain E and chain I of 1acb are predicted by each method; Two unbound structures are chain B of 2cga and the only one chain of 1egl.

**Table 6:** Detailed Results of DoBi, metaPPI, meta-PPISP and PPI-Pred on 27 targets

	**Protein 1**									**Protein 2**										
**Complex**		**DoBi**		**metaPPI**^**d**^		**meta-PPISP**^**d**^		**PPI-Pred**^**d**^			**DoBi**		**metaPPI**^**d**^		**meta-PPISP**		**PPI-Pred**			
	***Int***_**n**_^**a**^	***Acc***^**b**^	***Cov***^**c**^	***Acc***	***Cov***	***Acc***	***Cov***	***Acc***	***Cov***	***Int***_**n**_	***Acc***	***Cov***	***Acc***	***Cov***	***Acc***	***Cov***	***Acc***	***Cov***		
T01	11	46.2	54.5	—	—	83.3	62.5	—	—	13	38.9	53.8	—	—	0	0	—	—		
T02	7	24.1	100	—	—	72.2	43.3	—	—	6	21.4	100	—	—	0	0	—	—		
T03	10	12.0	30.0	—	—	60.0	75.0	—	—	15	32.0	53.3	—	—	19.6	18.0	—	—		
T04	19	50.0	89.5	0	0	58.3	38.9	2.4	3.6	18	37.5	100	64.3	40.9	0	0	71.4	68.2		
T05	20	29.2	35.0	0	0	52.6	33.3	4.8	9.1	17	14.3	35.3	90.0	39.1	4.5	7.7	38.9	30.4		
T06	23	28.6	34.8	71.4	29.4	39.1	27.3	59.5	73.5	29	38.1	27.6	28.6	15.4	25.8	66.7	4.5	3.8		
T07	15	52.9	60.0	33.3	30.8	33.3	30.8	0	0	11	15.4	18.2	7.7	5.6	5.6	4.3	0	0		
T08	25	37.9	44.0	0	0	9.5	8.3	0	0	23	64.0	69.6	30.0	11.5	0	0	7.9	11.5		
T09	37	90.5	51.4	80.0	20.0	0	0	25.8	20.0	37	76.7	62.2	45.5	12.5	0	0	16.1	12.5		
T10	46	40.0	47.8	—	—	10.0	47.4	—	—	53	50.0	49.1	—	—	0	0	—	—		
T11	12	50.0	91.7	86.7	59.1	—	—	45.8	50.0	28	71.9	82.1	81.8	50.0	—	—	56.5	72.2		
T12	12	16.7	25.0	93.8	62.5	61.5	30.8	45.5	41.7	28	86.4	67.9	55.6	33.3	36.0	45.0	22.2	13.3		
T13	10	33.3	100	—	—	0	0	—	—	8	44.4	100	—	—	72.0	85.7	—	—		
T14	53	52.2	22.6	10.0	2.3	6.8	33.3	8.6	7.0	63	42.3	17.5	50.0	13.2	13.5	19.2	2.0	2.6		
T15	23	95.0	82.6	0	0	63.2	50.0	5.0	11.1	19	81.0	89.5	15.8	33.3	56.5	72.2	9.1	11.1		
T16	—	—	—	55.6	21.7	87.0	74.1	0	0	—	—	—	100	29.0	25.0	53.8	61.8	67.7		
T17	—	—	—	0	0	23.1	12.5	0	0	—	—	—	92.9	65.0	0	0	33.3	45.0		
T18	24	53.6	62.5	85.7	50.0	42.9	36.0	46.2	50.0	31	50.0	35.5	0	0	52.2	36.4	2.1	3.4		
T19	12	68.8	91.7	—	—	33.3	28.0	—	—	12	45.0	75.0	—	—	69.2	62.1	—	—		
T20	47	53.6	31.9	94.4	37.8	23.8	90.9	28.6	22.2	35	72.2	37.1	72.2	36.1	34.3	54.5	23.2	63.9		
T21	17	73.7	82.4	0	0	0	0	3.0	6.7	15	55.6	66.7	0	0	33.3	20.8	0	0		
T22	17	22.7	29.4	9.1	6.7	28.6	17.4	0	0	12	71.4	83.3	83.3	41.7	6.2	5.9	60.0	75.0		
T23	49	95.6	87.8	64.3	17.0	18.2	53.3	66.0	62.3	49	95.3	83.7	64.3	17.0	0	0	66.0	62.3		
T24	3	13.3	66.7	66.7	66.7	—	—	50.0	73.3	1	5.6	100	0	0	—	—	50.0	61.5		
T25	—	—	—	100	68.2	20.0	23.5	81.8	81.8	—	—	—	58.3	31.8	73.9	77.3	55.6	90.9		
T26	34	43.8	41.2	75.0	27.3	20.8	33.3	0	0	24	61.5	66.7	21.4	12.5	18.2	60.0	18.2	8.3		
T27	7	43.8	87.5	0	0	0	0	6.7	22.2	8	50.0	91.7	20.0	22.2	0	0	0	0		

^a^*Int*_*n*_ is the number of residues on actual interface in complex.

^b^*Acc* (%) is the accuracy of the corresponding method on the data set.

^c^*Cov* (%) is the coverage of the corresponding method on the data set.

^d^The values for these methods are from literatures [10,14].

**Figure 3:**
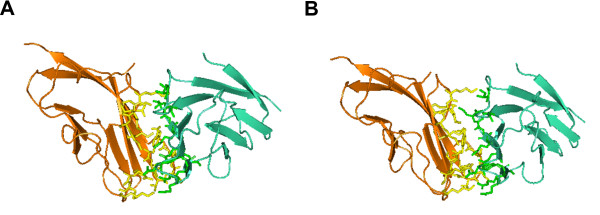
**Configuration discovered by DoBi for 1qa9(A:B).** (**A**) is the figuration by DoBi; and (**B**) is the experimental structure. The *C*_*α *_iRMSD between two complexes is 2.36Å.

Besides the identification of binding sites, our program also estimates the orientations and positions of the proteins after binding. Figure 3 displays the orientation and position discovered by our program for 1qa9(A:B). The *C*_*α*_ interface RMSD (root mean squared deviation) (iRMSD) between the experimental structure and the predicted complex is 2.36Å.

#### Comparison to ProMate and PINUP

In this experiment, DoBi is compared to ProMate and PINUP. The test data is originally used by ProMate, and consists of 57 non-homologous proteins. The results are reported in Table 7. DoBi has an F-score of 0.56, while PINUP and ProMate have the F-scores 0.43 and 0.21 respectively. The overall accuracy and coverage of DoBi are 54.2% and 59.1%. The success rate of DoBI is 64.9%. Hence the success rate is improved by at least 1.8%, while the overall accuracy and coverage are improved by at least 1.7% and 16.6% respectively.

**Table 7:** Comparison to PINUP and ProMate

	**DoBi**						**PINUP**						**ProMate**					
	**Suc**^**a**^	**Acc**^**b**^	**Cov**^**c**^	**F**^**f**^	***M***^**d**^	***V***^**e**^	**Suc**	**Acc**	**Cov**	**F**	***M***	***V***	**Suc**	**Acc**	**Cov**	**F**	***M***	***V***
Overall	64.9	54.2	59.1	0.56	23.5	10.5	42.1	44.9	42.5	0.43	19.0	8.7	63.1	52.5	13.2	0.21	5.4	16.8

^a^Suc (%) is the success rate of the corresponding method on the data set.

^b^Acc (%) is the average accuracy of the corresponding method on the data set.

^c^Cov (%) is the average coverage of the corresponding method on the data set.

^d^*M* is the average of the sizes predicted by the corresponding method on the data set.

^e^*V* is the standard deviation of the sizes predicted by the corresponding method on the data set.

^f^F is the F-score of the corresponding method on the data set.

The average of the sizes predicted by DoBi, PINUP and ProMate are 23.5 residues, 19.0 residues and 5.4 residues respectively, while the actual average size (average size of actual interface residues) is 21.0 residues. The number of residues correctly predicted to be on interface by DoBi, PINUP and ProMate are 12.3 residues, 8.3 residues and 2.7 residues respectively.

Table 8 shows the detailed results of 57 unbound proteins. DoBi performed better for most of the cases. However, for some cases where all three methods do not perform well, DoBi is usually the worst, e.g. 1avu_, 1aye_, 1qqrA and 1b1eA.

**Table 8:** Detailed Comparison to PINUP and ProMate

**PDB**^**a**^	**Complex**	***Int***_**n**_^**b**^	**DoBi**		**PINUP**^**g**^		**ProMate**^**f**^	
			**Acc**^**c**^	**Cov**^**d**^	**Acc**	**Cov**	**Acc**	**Cov**
1a19A	1brs(A:D)	16	86.7	81.3	72.2	81.3	100	29
1a2pA	1brs(D:A)	19	76.2	84.2	63.6	73.7	90	19
1a5e_	1bi7(B:A)	30	82.1	76.7	41.2	23.3	88	10
1acl_	1fss(A:B)	25	36.7	72.0	35.9	56.0	24	14
1ag6_	2pcf(A:B)	24	65.0	54.2	56.3	37.5	70	16
1aje_	1am4(D:A)	18	57.1	22.2	60.0	33.3	72	30
1ajw_	1cc0(E:A)	9	50.0	88.9	66.7	66.7	73	24
1aueA	1fap(B:A)	8	58.3	87.5	15.8	37.5	90	35
1avu_	1avw(B:A)	15	30.0	40.0	66.7	93.3	100	29
1aye_	1dtd(A:B)	22	42.1	36.4	44.4	54.5	54	24
1b1eA	1a4y(B:A)	32	38.7	37.5	88.2	46.9	69	24
1bip_	1tmq(B:A)	29	66.7	55.2	61.1	37.9	100	27
1ctm_	2pcf(B:A)	21	62.1	85.7	38.1	38.1	100	12
1cto_	1cd9(B:A)	6	40.0	33.3	35.3	100	36	29
1cye_	1eay(A:B)	16	55.6	62.5	5.6	6.3	0	0
1d0nA	1c0f(S:A)	27	46.2	44.4	0	0	67	3
1d2bA	1uea(B:A)	19	66.7	52.6	78.6	57.9	92	31
1ekxA	1d09(A:B)	21	64.5	95.2	0	0	0	0
1ex3A	1cgi(E:I)	33	61.1	33.3	68.2	45.5	100	29
1ez3A	1dn1(B:A)	18	88.9	44.4	47.1	44.4	100	6
1eza_	3eza(A:B)	21	64.0	76.2	0	0	0	0
1eztA	1agr(E:A)	22	57.1	54.5	22.2	18.2	54	13
1f00I	1f02(I:T)	17	31.6	35.3	0	0	0	0
1f5wA	1kac(B:A)	21	71.4	71.4	25.0	23.8	100	6
1fkl_	1b6c(A:B)	19	54.5	63.2	75.0	47.4	100	20
1flzA	1eui(A:C)	25	42.9	96.0	77.3	68.0	52	19
1fvhA	1dn1(A:B)	42	51.4	45.2	53.3	38.1	0	0
1g4kA	1uea(A:B)	30	46.2	40.0	43.8	23.3	78	21
1gc7A	1ef1(A:C)	18	71.4	55.6	28.6	11.1	78	6
1gnc_	1cd9(A:B)	15	43.7	46.7	21.4	20.0	6	2
1hh8A	1e96(B:A)	14	50.0	35.7	44.0	78.6	50	2
1hplA	1eth(A:B)	19	20.0	36.8	8.7	10.5	7	3
1hu8A	1ycs(A:B)	8	37.5	75.0	31.6	75.0	5	2
1iob_	1itb(A:B)	38	38.1	21.1	46.7	18.4	31	6
1j6zA	1c0f(A:S)	29	28.2	75.9	34.6	31.0	0	0
1jae_	1tmq(A:B)	32	60.0	65.6	83.3	46.9	50	13
1lba_	1aro(L:P)	16	8.6	18.8	40.0	37.5	60	24
1nobA	1kac(A:B)	15	50.0	73.3	0	0	7	3
1nos_	1noc(A:B)	9	33.3	44.4	0	0	0	0
1pco_	1eth(B:A)	15	77.8	46.7	16.7	20.0	60	12
1pne_	1hlu(P:A)	25	65.7	92.0	93.8	60.0	0	0
1poh_	1ggr(B:A)	10	57.1	40.0	72.7	80.0	0	0
1ppp_	1stf(E:I)	29	79.3	79.3	47.4	31.0	91	30
1qqrA	1bml(C:A)	7	33.3	28.6	38.5	71.4	85	32
1rgp_	1am4(A:D)	16	55.0	68.8	36.8	43.8	50	5
1selA	1cse(E:I)	29	75.0	93.1	60.9	48.3	61	27
1vin_	1fin(B:A)	29	40.0	34.5	50.0	51.7	0	0
1wer_	1wq1(G:R)	33	67.7	63.6	70.6	36.4	0	0
1xpb_	1jtg(A:B)	32	69.2	56.3	89.5	53.1	0	0
2bnh_	1a4y(A:B)	38	38.5	39.5	37.8	36.8	100	4
2cpl_	1ak4(A:D)	17	61.9	76.5	78.6	64.7	76	23
2f3gA	1ggr(A:B)	18	50.0	50.0	64.7	61.1	100	12
2nef_	1avz(B:A)	10	56.3	90.0	30.8	40.0	57	24
2rgf_	1lfd(A:B)	14	52.4	78.6	27.8	35.7	20	5
3ssi_	2sic(I:E)	15	80.0	80.0	68.2	100	100	24
6ccp_	2pcb(A:B)	9	23.5	44.4	28.6	66.7	0	0
Bound^e^	1jtg(B:A)	32	81.1	93.8	65.0	40.6	94	22

^a^PDB is the unbound structure of the predicted protein.

^b^*Int*_*n*_ is the number of residues on actual interface in complex.

^c^*Acc* (%) is the accuracy of the corresponding method on the data set.

^d^*Cov* (%) is the coverage of the corresponding method on the data set.

^e^The unbound structure of 1jtgB was not available in PDB, and we used the bound structure instead.

^f^The values for ProMate are from literature [9].

^g^The results for PINUP are calculated by using the same definition of actual interface with DoBi.

#### Comparison to core-SVM

In this study, we compare DoBi to core-SVM using the same data set of 50 dimers which core-SVM was tested against [12]. The results are shown in Table 9. The overall accuracy and coverage for our method are 59.0% and 61.1%, while those for core-SVM are 53.4% and 60.6%. The success rate of DoBi is 70.0% on 50 pairs of proteins in those binary complexes. The F-score is 0.60 for DoBi, and 0.56 for core-SVM. The average of the size predicted by DoBi is 39.0 residues (with standard deviation 19.1), while the average actual size is 40.3 residues. The number of residues correctly predicted by DoBi to be on the interface is 22.5.

**Table 9:** Comparison to core-SVM

	**DoBi**						**core-SVM**^**g**^					
	**Suc**^**a**^	**Acc**^**b**^	**Cov**^**c**^	**F**^**f**^	***M***^**d**^	***V***^**e**^	**Suc**	**Acc**	**Cov**	**F**	***M***	***V***
Overall	70.0	59.0	61.1	0.60	39.0	19.1	—	53.4	60.6	0.56	—	—

^a^*Suc* (%) is the success rate of the corresponding method on the data set.

^b^*Acc* (%) is the average accuracy of the corresponding method on the data set.

^c^*Cov* (%) is the average coverage of the corresponding method on the data set.

^d^*M* is the average predicted size for DoBi on the data set.

^e^*V* is the standard deviation of predicted size for DoBi on the data set.

^f^F is the F-score of the corresponding method on the data set.

^g^The values for core-SVM are from literature [12].

Table 10 shows the details for DoBi on the data set used by core-SVM. The performance of DoBi is particularly good on several proteins such as 1aym2 and 1rzhM.

**Table 10:** Detailed Results for DoBi on the data set used by core-SVM

**Protein ID**	**Partner ID**	***Int***_**n**_^**a**^	***C***_***n***_^**b**^	***P***_***n***_^**c**^	**Acc**^**d**^	**Cov**^**e**^
1a9xA	1a9xB	59	52	95	54.7	88.1
1a9xB	1a9xA	52	47	88	53.4	90.4
1aym1	1aym3	46	38	41	92.7	82.6
1aym2	1aym1	57	54	70	77.1	94.7
1aym3	1aym1	43	33	36	91.7	76.7
1blxA	1blxB	21	15	33	45.5	71.4
1fzcB	1fzcC	45	38	58	65.5	84.4
1g4yR	1g4yB	29	5	18	27.8	17.2
1gk8A	1gk8I	49	28	55	50.9	57.1
1h1rB	1h1rA	33	9	14	64.3	27.2
1h8eC	1h8eD	69	37	67	55.2	53.6
1h8eD	1h8eC	35	19	39	48.7	54.3
1hxs4	1gxs1	31	21	35	60.0	67.7
1irdB	1irdA	23	20	32	62.5	86.9
1j34A	1j34B	43	19	22	86.4	44.1
1jboB	1jboA	36	16	29	55.2	44.4
1jsdA	1jsdB	51	18	20	90.0	35.3
1jsdB	1jsdA	67	26	42	61.9	38.8
1k5nA	1k5nB	35	24	56	42.9	68.6
1k5nB	1k5nA	25	16	39	41.0	64.0
1ld8A	1ld8B	35	23	28	82.1	65.7
1mtyB	1mtyD	58	22	34	64.7	38.1
1mtyD	1mtyB	31	10	15	66.7	32.2
1mtyG	1mtyD	41	18	42	42.9	43.9
1n4qB	1n4qA	25	5	15	33.3	20.0
1p2jA	1p2jI	23	18	36	50.0	78.2
1p2jI	1p2jA	14	13	21	61.9	92.9
1qopA	1qopB	35	32	52	61.5	91.4
1qopB	1qopA	34	31	51	60.8	91.2
1rthA	1rthB	57	32	68	47.0	56.1
1rthB	1rthA	58	33	69	47.8	56.9
1rypB	1rypA	31	13	24	54.1	41.9
1rzhH	1rzhM	37	8	16	50.0	21.6
1rzhL	1rzhM	48	42	45	93.3	87.5
1rzhM	1rzhL	51	45	48	93.8	88.2
1s5dD	1s5dA	4	4	29	13.7	100
1tugA	1tugB	17	14	39	35.9	82.4
1tugB	1tugA	12	9	24	37.5	75.0
1tx4B	1tx4A	25	18	34	52.9	72.0
1uvqA	1uvqB	61	35	39	89.7	57.4
1uvqB	1uvqA	55	26	31	83.9	47.2
1we3F	1we3T	12	10	48	20.8	83.3
1wf4o	1wf4a	10	10	19	52.6	100
2ltnA	2ltnB	55	12	16	75.0	21.8
2ltnB	2ltnA	47	17	17	100	36.2
3pcgA	3pcgM	41	12	15	80.0	29.3
3pcgM	3pcgA	40	11	21	52.4	27.5
4ubpA	4ubpC	24	8	43	18.6	33.3
4ubpC	4ubpB	46	26	86	30.2	56.5
8rucI	8rucA	38	29	38	76.3	76.3

^a^*Int*_*n*_ is the number of residues on actual interface in complex.

^b^*C*_*n*_ is the number of residues correctly predicted to be on interface by our method.

^c^*P*_*n*_ is the number of total residues predicted to be on interface by our method.

^d^Acc (%) is the accuracy of our method on the data set.

^e^Cov (%) is the coverage of our method on the data set.

### Evaluation on benchmark v4.0

To further evaluate our method, we perform tests on the protein-protein docking benchmark v4.0 [32,33]. This benchmark consists of 176 complexes. Proteins dynamically change their conformations upon binding with other proteins [34]. A single protein without binding with any other structure is referred to as *unbound*, whereas a protein with a binding partner in a complex is referred to as *bound*. We test our method in both the bound and the unbound cases. 

"Running time"

We used a Pentium(R) 4 (CPU of 3.40GHz) to run DoBi. The computation for each of the 176 complexes took 100 seconds on average.

#### Results on bound states

The complexes are classified into broad biochemical categories: Enzyme-Inhibitor (52), Antibody-Antigen (25) and Others (99). The average accuracy and coverage of DoBi are 61.8% and 67.9% respectively on the 52 complexes in Enzyme-Inhibitor, 51.6% and 70.1% on the 25 complexes in Antibody-Antigen, and 58.2% and 69.1% on the 99 complexes in Others. A success rate of 77.6% is achieved for the Enzyme-Inhibitor complexes. The details are shown in Table 11.

**Table 11:** DoBi’s performance for proteins of benchmark v4.0 in bound states

**Type**^**a**^	**No. of complexes**	**Suc**^**b**^	**Acc**^**c**^	**Cov**^**d**^	***M***^**e**^	***V***^**f**^
Enzyme-Inhibitor	52	77.6	61.8	67.9	22.6	6.3
Antibody-Antigen	25	56.0	51.6	70.1	19.3	6.5
Others	99	66.7	58.2	69.1	24.0	10.8
Overall	176	68.2	57.5	68.9	22.9	9.3

^a^Type is based on the broad biochemical categories.

^b^Suc (%) is the success rate of DoBi on the data set.

^c^Acc (%) is the average accuracy of DoBi on the data set.

^d^Cov (%) is the average coverage of DoBi on the data set.

^e^*M* is the average of the sizes predicted by DoBi on the data set.

^f^*V* is the standard deviation of the sizes predicted by DoBi on the data set.

#### Results on unbound states

The pairs of unbound proteins are classified into three categories: 121 rigid-body (easy) cases, 30 medium difficult cases, and 25 difficult cases, according to the magnitude of conformational change after binding [30]. The average accuracy and coverage of DoBi are 43.6% and 65.4% on the 121 rigid-body cases, 34.1% and 56.7% on the 30 medium difficult cases, and 32.4% and 53.4% on the 25 difficult cases. The success rate of DoBi is 41.7% for the rigid-body cases, which is significantly better than for the other two categories. In general, the accuracy and coverage decrease as the magnitude of conformational increases. The details are shown in Table 12.

**Table 12:** DoBi’s performance for proteins of benchmark v4.0 in unbound states

**Subset**^**a**^	**Type**^**b**^	**No. of cases**	**Suc**^**c**^	**Acc**^**d**^	**Cov**^**e**^	***M***^**f**^	***V***^**g**^
Rigid body	Enzyme-Inhibitor	40	51.2	48.9	66.9	37.1	34.1
	Antibody-Antigen	22	50.0	51.0	67.8	24.0	14.6
	Others	59	32.2	37.3	63.5	39.9	36.9
	Subtotal	121	41.7	43.6	65.4	36.1	31.9
Medium difficult	Enzyme-Inhibitor	7	39.9	36.7	56.2	25.9	17.4
	Antibody-Antigen	1	0	31.9	41.4	38.0	9.2
	Others	22	31.2	33.4	56.7	52.9	56.7
	Subtotal	30	31.6	34.1	56.7	46.1	45.9
Difficult	Enzyme-Inhibitor	5	37.5	43.1	46.5	26.1	7.0
	Antibody-Antigen	2	0	29.5	54.6	27.3	17.5
	Others	18	10.5	30.5	54.8	54.9	44.8
	Subtotal	25	13.9	32.4	53.4	46.9	35.1
Overall		176	36.0	40.4	62.2	39.3	36.9

^a^Subset is based on the magnitude of conformational change after binding.

^b^Type is based on the broad biochemical categories.

^c^Suc (%) is the success rate of DoBi on the data set.

^d^Acc (%) is the average accuracy of DoBi on the data set.

^e^Cov (%) is the average coverage of DoBi on the data set.

^f^*M* is the average predicted size for DoBi on the data set.

^g^*V* is the standard deviation of predicted size for DoBi on the data set.

**Figure 4:**
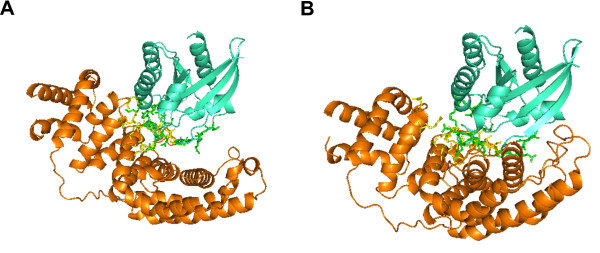
**Configuration discovered by DoBi for 1wq1(R:G).** (**A**) is the configuration by DoBi; and (**B**) is the experimental structure. The *C*_*α *_iRMSD between two complexes is 4.12Å.

DoBi discovered several good configurations for the medium difficult cases. One of the instances is 1wq1(R:G). Its configuration discovered by DoBi is shown in Figure 4. The *C*_*α*_ iRMSD between the experimental structure and the predicted complex is 4.12Å.

### Docking result of DoBi

DoBi is optimized for binding site prediction, but it also can be used to dock two protein structures. We compare DoBi’s poses to the best configurations obtained by ZDOCK and 3D-Dock. ZDOCK [35] uses a fast Fourier transform to search all possible binding modes for the proteins, and evaluates them based on shape complementarity, desolvation energy, and electrostatics. It can produce structures with the smallest iRMSD values in top 1000 predictions with minimum energy. 3D-Dock [36,37] uses an initial grid-based shape complementarity search to produce lots of potential interacting conformations. They can be ranked by using interface residue propensities and interaction energies. It reports structures with the smallest iRMSD values in top ten predictions.

**Table 13:** The Docking Results of DoBi, ZDOCK and 3D-Dock on CAPRI

**Target**	**DoBi**_**1000**_				**ZDOCK**				**DoBi**_**10**_				**3D-Dock**^**e**^	
	**iRMSD**^**a**^	**NC**^**b**^	**F**_***l***_^**c**^	**F**_***r***_^**d**^	**iRMSD**	**NC**	**F**_***l***_	**F**_***r***_	**iRMSD**	**NC**	**F**_***l***_	**F**_***r***_	**iRMSD**	**NC**
T1	4.28	27.6	56.0	45.2	8.10	17.2	50.0	32.0	5.45	44.0	74.1	64.3	3.0	46
T2	6.23	76.9	38.8	35.3	4.15	46.2	51.9	35.7	8.27	53.8	48.0	36.4	—	—
T3	18.48	9.4	17.1	43.9	3.89	62.5	64.0	60.6	18.51	12.0	22.9	51.4	—	—
T4	3.98	63.5	66.6	57.1	4.50	23.1	78.2	58.3	6.24	35.9	38.3	51.6	15.1	21
T5	11.06	7.7	46.8	31.6	10.08	5.4	76.6	18.9	11.06	7.7	46.8	31.6	—	—
T6	16.49	15.4	36.4	33.4	8.72	29.2	54.2	71.6	19.21	9.6	18.2	28.1	0.8	86
T7	11.10	13.5	62.8	24.0	6.43	2.7	44.4	4.8	11.10	13.5	62.8	24.0	28.6	14
T8	6.69	37.9	42.7	60.9	2.73	63.6	82.8	60.0	6.69	37.9	42.7	60.9	1.7	33
T9	2.85	33.3	61.3	67.6	8.46	28.9	54.1	58.7	10.54	1.4	36.7	37.7	9.7	23
T10	4.52	28.9	50.4	51.8	14.75	5.9	15.4	17.3	7.69	13.0	58.1	59.3	34.8	0
T11	2.55	66.7	68.5	75.0	2.63	61.1	96.0	82.1	12.17	0	0	45.0	1.9	20
T12	2.55	66.7	68.5	75.0	2.31	81.5	75.9	88.9	12.17	0	0	45.0	3.2	22
T13	3.33	94.1	74.1	69.6	2.49	57.1	52.9	59.3	3.33	94.1	74.1	69.6	6.4	6
T14	19.98	9.6	34.5	28.0	5.22	42.0	72.7	68.9	20.97	10.3	36.1	28.3	0.9	47
T15	2.40	53.6	86.9	83.0	0.86	91.1	90.6	81.8	4.00	42.0	64.2	63.6	—	—
T18	8.08	25.0	57.7	44.4	1.88	66.2	80.0	80.0	11.38	8.2	10.3	19.7	9.4	14
T19	2.74	58.8	60.0	69.0	9.81	4.8	40.0	14.6	2.74	58.8	60.0	69.0	3.9	31
T20	15.13	1.1	14.7	28.6	13.62	7.2	35.0	37.1	15.13	1.1	14.7	28.6	—	—
T21	2.02	50.0	77.8	68.8	2.43	70.7	83.3	70.6	2.02	50.0	77.8	68.8	—	—
T22	16.08	7.5	20.0	71.4	9.28	12.6	66.7	0	16.08	7.5	20.0	71.4	—	—
T23	1.90	61.2	86.9	88.4	2.14	72.1	87.3	87.9	3.14	46.0	83.1	83.1	—	—
T24	5.01	50.0	31.6	20.0	28.15	0	0	0	5.01	50.0	31.6	20.0	—	—
T26	7.11	29.6	26.1	45.2	30.07	0	0	0	7.11	29.6	26.1	45.2	—	—
T27	6.95	60.0	42.4	51.9	15.89	3.5	24.4	0	7.38	66.7	38.5	50.0	—	—
T29	2.46	68.6	83.3	79.3	3.90	58.6	77.4	72.1	3.80	32.7	69.4	77.8	—	—

^a^*C*_*α *_iRMSD between the configuration by the respective method and the experimental structure.

^b^*NC* (%) is fraction of native contacts for each method.

^c^F_*l*_(%) is the F-score of each method for the ligand protein on the data set.

^d^F_*r *_(%) is the F-score of each method for the receptor protein on the data set.

^e^The values for 3D-Dock are from literatures [36,37]; The blank results mean that 3D-Dock never produced on these targets.

We calculate the predicted structures by different methods on the complexes in benchmark v4.0 and the targets in CAPRI. CAPRI is a community-wide experiment to assess the capacity of protein docking methods to predict protein-protein interactions [31]. The *C*_*α *_iRMSD, F-score and the fraction of native contacts are used to evaluate the results by different methods. The fraction of native contacts is used by 3D-Dock [37]. It is calculated as the total number of native contacts for the predicted configuration divided by the total number of contacts in the native structure. A native contact exists between residues i and j if distances between them in native structure and in predicted configuration are both less than 4.5Å.

We compare the docking results of DoBi, ZDOCK and 3D-DOCK on the CAPRI targets. The results are shown in Table 14. The top 1,000 configurations predicted by DoBi and ZDOCK are used for comparison. Among the 1,000 predictions, we choose the configuration of the best iRMSD value to evaluate the methods. The average iRMSD values for DoBi and ZDOCK are 7.5Å and 6.9Å, respectively. However, the average fractions of native contacts for DoBi and ZDOCK are 40.6% and 35.2%, respectively. DoBi improves the F-score of binding site prediction by at least 1.3%. DoBi’s performance on docking is worse than ZDOCK, but its performance on binding site prediction is more accurate than ZDOCK.

**Table 14:** The Docking Results of DoBi and ZDOCK on Benchmark v4.0

	**DoBi**_**1000**_			**ZDOCK**				**DoBi**_**1000**_			**ZDOCK**				**DoBi**_**1000**_			**ZDOCK**		
**PDB**	**iRmsd**^**a**^	**F**_***l***_^**b**^	**F**_***r***_^c^	**iRmsd**	**F**_***l***_	**F**_***r***_	**PDB**	**iRmsd**	**F**_***l***_	**F**_***r***_	**iRmsd**	**F**_***l***_	**F**_***r***_	**PDB**	**iRmsd**	**F**_***l***_	**F**_***r***_	**iRmsd**	**F**_***l***_	**F**_***r***_
1bvk	1.24	71.8	72.7	1.72	71.4	80.0	1jps	4.27	66.7	62.8	2.26	78.3	82.6	1gla	6.51	77.3	72.4	3.76	70.3	72.0
2sni	1.49	92.9	82.8	2.55	90.0	78.3	1yvb	4.44	71.0	51.3	1.61	82.4	91.3	1acb	6.55	78.8	78.0	2.61	93.8	82.6
1j2j	1.52	80.0	83.9	2.18	66.7	56.4	1avx	4.54	66.7	70.2	1.67	73.3	88.5	2i25	6.57	46.2	68.6	1.40	80.0	72.0
1wq1	1.60	88.5	76.9	1.82	77.6	69.2	1fq1	4.54	62.9	76.5	8.05	42.4	50.0	1z0k	6.60	72.7	55.2	2.29	90.3	75.0
1rv6	1.68	80.0	88.2	1.43	86.7	83.3	1e6e	4.58	67.9	60.9	1.11	85.0	85.7	1fc2	6.88	59.5	73.7	3.53	69.0	58.1
1z5y	1.70	82.9	89.5	1.69	85.7	86.4	2cfh	4.58	63.8	66.7	1.53	84.2	76.6	1oph	6.97	72.2	80.8	2.00	70.6	58.1
1n8o	1.73	81.5	89.9	2.28	82.9	78.7	1oyv	4.61	85.7	66.7	2.12	83.0	84.1	1jmo	7.01	80.0	66.6	18.99	36.4	0
1buh	1.98	82.4	70.3	1.12	87.5	96.3	1kkl	4.74	36.4	57.9	27.92	0	0	1he1	7.07	90.0	89.7	2.02	80.9	70.6
2j0t	2.16	57.1	48.8	4.86	59.1	56.1	1bvn	4.82	74.5	48.0	1.72	87.5	82.9	1xd3	7.08	66.7	72.2	0.45	96.3	93.8
1qa9	2.20	47.6	61.1	4.00	51.9	64.5	1gp2	4.83	86.1	84.4	3.39	56.2	92.9	2oor	7.17	64.5	68.7	3.14	75.0	63.0
1gcq	2.27	90.9	75.3	5.19	71.0	64.0	1ktz	4.84	80.0	69.6	3.68	91.7	63.6	1ibr	7.23	55.3	63.4	9.83	50.6	33.8
1b6c	2.32	71.0	77.8	2.63	82.9	88.4	2g77	4.84	68.1	61.6	1.52	94.5	86.2	1ak4	7.25	52.2	52.6	4.28	85.7	90.0
2b42	2.35	78.6	88.2	1.36	94.1	87.7	2btf	4.86	71.7	66.7	2.48	74.4	80.0	1vfb	7.27	48.9	50.0	2.30	74.3	72.2
2a5t	2.38	82.1	78.8	4.36	52.0	40.0	1jiw	4.86	79.5	81.5	5.22	56.5	66.7	1k4c	7.29	70.3	44.4	1.47	81.2	97.7
1gpw	2.45	79.1	64.0	1.51	81.6	78.4	1gxd	4.88	73.3	62.5	3.41	80.9	64.9	2vdb	7.31	74.1	64.8	1.28	90.5	100
1fle	2.47	78.6	73.2	4.01	74.1	44.0	1f51	4.89	70.6	68.6	2.40	66.7	68.3	1gl1	7.42	96.3	86.8	1.55	81.2	83.3
2ido	2.48	87.5	82.8	5.09	71.4	80.0	1jzd	4.92	75.0	71.0	2.67	76.2	73.7	1syx	7.49	75.0	75.7	4.81	64.5	85.0
1fqj	2.49	79.1	66.7	13.13	16.7	26.3	1pvh	4.94	54.5	79.0	1.92	75.0	88.9	1eer	7.49	66.7	53.8	7.90	58.1	54.5
2hrk	2.51	100	88.2	2.06	80.0	70.6	1m10	4.96	75.9	60.0	9.42	36.1	29.8	2oob	7.58	53.3	71.0	5.38	81.8	81.8
1dqj	2.52	79.2	91.3	8.31	53.7	35.9	2abz	4.99	54.8	58.6	3.73	89.7	84.6	1jtg	7.69	78.1	76.7	1.39	81.5	80.7
1ezu	2.53	84.7	74.7	2.38	94.3	78.9	1bkd	5.04	81.1	77.1	7.33	59.6	53.5	1nsn	7.91	73.9	74.4	4.82	42.1	82.1
1k5d	2.54	90.4	78.4	2.51	73.0	70.0	1i2m	5.06	85.7	64.5	2.21	77.4	83.6	1zm4	7.98	43.6	31.4	2.44	66.7	56.0
2qfw	2.61	93.3	87.5	1.58	88.9	73.7	1e6j	5.06	54.1	50.0	1.57	100	100	1udi	8.08	51.1	50.0	1.42	88.9	86.7
2ayo	2.61	73.4	68.9	1.85	92.6	88.9	3sgq	5.09	81.8	77.3	2.19	84.4	84.4	2ot3	8.11	76.3	71.6	4.40	64.2	73.7
2hle	2.63	55.6	58.8	3.52	72.7	61.2	1ewy	5.13	65.0	66.7	2.47	73.2	77.8	3cph	8.29	73.2	59.3	3.91	66.7	66.7
1zhh	2.67	66.7	70.4	9.28	27.5	45.6	1kxp	5.13	62.0	78.8	2.00	80.0	66.7	1eaw	8.31	95.2	85.3	1.34	92.9	92.3
1ay7	2.73	74.3	61.5	4.64	66.7	40.7	2c0l	5.14	84.9	71.8	4.36	45.7	41.9	1tmq	8.53	57.2	61.6	2.42	90.6	82.8
1f6m	2.76	84.0	83.3	12.24	26.1	19.2	2hmi	5.14	60.0	46.1	26.99	73.9	0	1efn	8.61	66.7	64.3	6.62	63.6	41.7
2a9k	2.80	89.7	81.0	5.67	62.1	37.8	1pxv	5.17	83.9	86.5	3.81	61.9	62.7	1n2c	8.66	86.4	78.9	3.21	75.7	92.8
1oc0	2.82	77.4	57.2	2.95	75.9	75.9	1sbb	5.18	75.9	76.9	8.23	21.4	37.8	2fju	8.75	76.6	60.0	1.47	81.5	81.5
1i4d	2.97	71.1	65.3	1.97	68.4	64.9	1us7	5.18	55.6	76.2	1.17	88.0	84.6	1r0r	8.91	76.5	59.0	2.10	80	82.4
2o8v	2.97	66.6	57.1	2.76	84.2	66.6	2jel	5.25	80.0	77.3	2.40	93.3	79.1	1wej	8.96	42.9	53.3	24.79	5.7	0
1wdw	3.02	73.8	70.2	1.54	94.6	87.5	1fcc	5.25	66.7	47.6	11.33	29.4	32.5	1s1q	9.13	93.3	88.9	1.76	97.0	72.7
1mq8	3.02	71.8	66.7	6.72	85.7	29.6	1lfd	5.27	62.9	50.0	4.94	70.0	64.3	2o3b	9.15	48.0	48.6	14.16	44.4	32.0
2z0e	3.02	75.6	80.0	4.24	69.6	58.2	2j7p	5.38	66.7	64.7	6.89	50.9	59.0	1e4k	9.42	75.9	86.2	15.2	21.7	12.8
1nw9	3.02	70.6	66.7	3.19	78.8	68.6	1akj	5.44	66.7	79.3	5.55	61.5	74.1	1cgi	9.48	80.0	77.4	1.59	97.4	89.3
1ofu	3.13	66.7	82.4	2.05	81.2	84.8	1ijk	5.46	60.6	44.5	1.86	91.7	74.3	1clv	9.48	77.1	66.6	1.58	88.9	87.0
1i9r	3.20	62.1	59.2	21.89	37.5	0	2nz8	5.49	72.8	75.0	2.87	82.6	75.8	7cei	9.51	54.5	48.5	0.88	88.0	88.9
1e96	3.21	96.5	84.6	2.98	55.2	63.2	1h9d	5.49	62.9	80.0	1.88	84.4	81.1	2vis	9.57	81.0	91.4	22.23	0	0
1t6b	3.22	71.2	70.2	1.19	85.0	90.9	1rlb	5.50	76.2	58.8	14.71	26.7	23.8	1bgx	9.90	78.3	80.0	11.09	50.5	27.2
2oul	3.30	64.9	65.5	1.97	80.0	86.8	1bj1	5.59	83.3	85.7	2.11	92.7	88.0	1d6r	10.54	41.0	66.6	12.68	25.0	22.9
1ahw	3.36	45.2	57.1	1.86	88.9	89.4	1r6q	5.59	50.0	78.6	5.20	47.4	53.3	2ajf	10.60	52.4	51.1	3.57	72.3	69.6
1y64	3.44	94.1	88.9	15.37	31.6	21.6	1qfw	5.61	48.0	57.2	1.50	93.3	77.8	1ml0	10.69	54.0	42.4	1.29	82.6	86.8
1ffw	3.44	42.1	66.7	3.91	56.0	57.1	2uuy	5.66	66.7	62.3	4.20	44.5	76.2	1k74	10.73	39.1	20.2	1.63	76.6	80.8
1grn	3.46	78.8	70.3	1.81	69.4	70.0	1iqd	5.66	64.9	70.4	1.26	94.4	80.0	1dfj	11.14	48.3	35.5	1.29	87.9	82.4
2pcc	3.52	65.4	66.7	5.34	76.5	43.3	2oza	5.71	60.5	69.2	8.49	40.8	28.9	1kac	11.24	74.1	42.9	3.22	87.8	85.0
1hcf	3.57	75.9	71.4	0.95	90.9	86.5	1fak	5.73	71.4	86.3	7.73	40.0	44.9	1xu1	11.36	87.5	78.8	1.54	89.7	80.0
1a2k	3.57	75.7	50.0	1.91	55.8	53.7	1de4	5.76	53.9	70.0	1.77	80.0	78.4	1mah	11.55	86.9	73.5	1.87	86.5	83.6
1jwh	3.61	66.7	75.6	1.28	80.0	66.7	1zgi	5.82	90.9	88.2	1.79	78.3	85.7	1he8	11.95	58.3	56.3	2.38	60.0	64.3
1atn	3.70	72.3	83.3	4.74	79.1	80.0	1azs	5.86	62.9	75.9	1.18	84.2	83.3	1fsk	11.99	61.1	62.8	1.15	91.9	90.5
2sic	3.76	72.2	76.4	0.94	96.3	90.9	1hia	5.91	66.7	56.1	12.4	23.0	28.6	1h1v	14.13	20.4	38.9	16.72	18.2	20.7
1ppe	3.83	76.9	83.3	1.42	86.7	93.5	1mlc	6.18	54.2	71.9	1.52	80.0	78.9	1xqs	14.27	37.2	23.3	1.67	79.1	85.7
1klu	3.94	83.7	90.9	11.1	27.5	43.2	2fd6	6.20	63.0	53.3	4.34	75.9	43.4	1jk9	15.43	84.4	74.4	2.16	82.9	73.2
1zli	3.97	87.1	71.4	12.25	43.2	28.6	4cpa	6.21	62.1	72.0	1.74	80.0	81.0	1ghq	16.12	68.3	57.7	22.15	0	48.0
3d5s	3.97	70.0	72.3	2.08	81.1	84.2	1nca	6.25	64.5	71.0	1.38	90.2	87.0	1r8s	20.83	12.6	57.7	6.48	49.2	54.5
2b4j	4.00	82.4	83.7	10.33	35.7	28.6	1f34	6.36	59.3	61.5	1.94	84.8	83.6	1kxq	21.12	75.0	80.0	1.18	84.6	93.5
2i9b	4.18	80.0	79.2	5.58	78.3	42.1	1ib1	6.42	64.8	71.4	5.89	53.1	46.4	2hqs	26.33	10.5	22.6	12.37	15.0	29.1
2mta	4.18	82.1	86.5	1.64	84.6	82.4	3bp8	6.49	61.0	63.2	4.02	57.9	68.8	1ira	28.13	31.8	25.0	16.42	36.5	28.9
2h7v	4.19	72.2	81.2	2.64	85.7	80.0														

^a^*C*_*α *_iRMSD between the configuration by methods and the experimental structure.

^b^F_*l*_ (%) is the F-score of each method for the ligand protein on the data set.

^c^F_*r*_ (%) is the F-score of each method for the receptor protein on the data set.

Each of DoBi and 3D-Dock produced ten results for each target, and the configurations with smallest iRMSD values among those ten predictions are used for comparison. The average iRMSD values for DoBi and 3D-Dock are 9.2Å and 9.1Å. However, the overall fractions of native contacts for DoBi and 3D-Dock are 29.1% and 26.8%. DoBi’s performance on binding site prediction is better than that of 3D-Dock.

The docking results obtained by DoBi and ZDOCK on Benchmark v4.0 are shown in Table 15. Similarly, we compare the best configurations in the top 1000 predictions from each method of DoBi and ZDOCK for each target. The average iRMSD values of DoBi and ZDOCK are 6.1Å and 4.9Å, respectively. For the binding site prediction, the overall F-score values of ligand proteins by DoBi and ZDOCK are 69.5% and 69.4%, and those of receptor proteins by DoBi and ZDOCK are 68.2% and 66.1%, respectively. These results indicate that DoBi’s performance on binding site prediction is better than ZDOCK. The docking quality of DoBi requires further efforts to improve.

**Table 15:** Comparison of Atomic Contact Energy for the Predicted Complexes and the Experimental Structures on Benchmark v4.0

**PDB**	**E**_***act***_^**a**^	**E**_***pre***_^**b**^	**F**_***r***_^**c**^	**F**_***l***_^**d**^	**PDB**	**E**_***act***_	**E**_***pre***_	**F**_***r***_	**F**_***l***_	**PDB**	**E**_***act***_	**E**_***pre***_	**F**_***r***_	**F**_***l***_	**PDB**	**E**_***act***_	**E**_***pre***_	**F**_***r***_	**F**_***l***_
2o8v(A:B)	-96.7	-149.3	91.4	81.0	1a2k(C:B)	-38.9	-314.8	72.7	71.8	2vis(B:C)	35.8	-389.7	62.8	61.1	1n2c(A:F)	97.6	-272.1	46.1	60.0
1hcf(A:X)	-46.2	-13.4	90.9	83.7	4cpa(A:I)	-47.6	-318.3	72.7	71.0	1acb(E:I)	-157.4	-555.7	62.2	92.9	1xu1(A:T)	60.7	85.9	45.5	72.7
1z5y(D:E)	-85.9	-51.1	89.7	90.0	1wq1(R:G)	161.0	306.0	72.4	77.3	1j2j(A:B)	-49.1	-161.8	62.1	51.6	1gpw(A:B)	107.8	-144.5	45.0	75.7
1gcq(B:C)	-5.3	4.4	88.9	94.1	1mah(A:F)	-8.5	-303.0	72.4	64.3	1jzd(A:C)	55.2	78.5	61.6	57.1	1oyv(B:I)	-70.5	-136.0	44.5	60.6
2j0t(A:D)	-65.8	-74.5	88.9	93.3	1udi(E:I)	69.2	-93.0	72.0	50.0	2hmi(D:B)	-7.1	-524.8	61.6	57.2	1d6r(A:I)	88.0	-66.6	43.4	66.7
1s1q(A:B)	20.4	168.1	88.2	90.9	1t6b(X:Y)	87.4	-675.9	71.9	54.2	1vfb(B:C)	81.7	191.4	61.5	59.3	2fju(B:A)	61.0	-658.9	43.1	27.9
2ayo(A:B)	340.9	384.2	86.8	96.3	2g77(A:B)	215.5	72.6	71.6	76.3	1b6c(A:B)	-29.8	-130.3	61.1	47.6	2sic(E:I)	-158.8	-321.2	42.9	74.1
1n8o(C:E)	-91.8	-64.1	86.5	83.9	1wdw(B:A)	301.6	30.6	71.4	64.8	1oph(A:B)	-10.1	-516.1	60.6	64.5	1eer(A:B)	143.2	219.2	42.1	71.4
1i4d(D:A)	30.0	-49.1	86.5	82.1	1jk9(B:A)	1.7	-150.6	71.4	69.9	1f51(A:E)	179.1	37.4	60.0	47.4	1ofu(X:A)	-32.7	-193.5	41.8	62.9
1qa9(A:B)	260.0	-73.9	85.7	83.3	1gp2(A:B)	56.5	-48.6	71.4	72.7	2i25(N:L)	131.0	144.8	60.0	76.6	2abz(B:E)	34.0	-300.7	41.0	70.3
2hle(A:B)	83.0	179.3	85.3	95.2	1dqj(B:C)	119.4	104.1	71.4	71.4	1ktz(A:B)	-24.0	-150.3	60.0	57.2	1fq1(A:B)	152.5	-187.8	40.8	30.8
1fle(E:I)	-134.1	-248.0	84.6	96.5	1us7(A:B)	71.6	30.8	71.0	64.5	1i9r(H:A)	92.9	284.5	60.0	58.8	1jps(H:T)	258.6	366.2	37.8	66.7
1jtg(B:A)	232.8	257.6	84.4	86.1	1kkl(A:H)	105.2	-252.2	71.0	75.0	1sbb(A:B)	0.5	154.1	60.0	58.8	1xqs(A:C)	368.9	383.0	37.2	23.3
1hia(B:I)	-4.8	51.3	83.7	56.0	3d5s(A:C)	89.8	-70.8	70.8	68.3	1ffw(A:B)	79.2	68.9	59.2	62.1	1zm4(A:B)	118.6	-236.7	35.8	33.3
1k5d(A:C)	197.6	305.1	81.5	79.5	1r6q(A:C)	-71.5	-129.3	70.4	64.9	2i9b(E:A)	58.0	-87.2	59.0	76.5	1ijk(C:A)	85.1	-45.5	35.7	14.8
1yvb(A:I)	-141.9	-271.2	80.8	72.2	1he1(C:A)	20.6	242.2	70.3	66.6	1pxv(A:C)	28.5	-79.4	58.8	76.2	1tmq(A:B)	2.1	-466.6	35.1	63.0
1fak(L:T)	108.7	217.2	80.0	75.6	1rv6(V:X)	-17.7	-3.7	70.0	52.6	1r0r(E:I)	-126.0	-127.4	58.8	61.5	2ido(A:B)	-71.8	92.8	33.4	39.0
3sgq(E:I)	-57.9	26.3	80.0	75.0	1bkd(R:S)	195.0	-49.0	69.6	78.6	1e6j(H:P)	14.9	-366.7	58.8	40.0	1oc0(A:B)	27.1	-417.1	33.3	66.7
1pvh(A:B)	121.5	13.5	80.0	62.9	1avx(A:B)	31.8	35.1	69.2	81.5	1jmo(A:H)	-49.0	-492.6	57.9	32.5	1y64(A:B)	123.8	-239.0	32.3	36.0
2oob(A:B)	-15.8	-28.9	80.0	78.3	1zhi(A:B)	93.8	-89.3	68.7	64.5	3cph(G:A)	84.4	-193.3	57.7	68.3	1dfj(E:I)	159.3	-394.3	32.1	44.4
1oyv(A:I)	-152.8	-158.1	79.3	66.7	1kac(A:B)	92.6	66.9	68.6	57.9	1ewy(A:C)	55.6	-80.1	57.2	63.1	1m10(A:B)	168.3	-36.2	31.8	54.1
1i2m(A:B)	300.9	213.4	79.2	79.4	1gl1(A:I)	-83.2	-282.7	68.1	78.8	2h7v(A:C)	67.5	9.9	57.2	73.2	1ira(Y:X)	212.7	48.1	31.8	25.0
1atn(A:D)	-72.3	-365.5	79.1	73.3	1e6e(A:B)	246.8	-137.5	67.7	73.4	1qfw(M:B)	60.9	61.7	57.2	48.0	2oul(A:B)	-123.9	-311.2	30.4	54.0
1klu(A:D)	60.2	-243.4	79.0	54.5	1bj1(H:W)	10.9	-139.6	66.7	71.0	2z0e(A:B)	-38.7	-562.7	56.4	63.8	1k74(A:D)	127.4	145.5	30.0	27.3
2hrk(A:B)	-5.4	-52.5	78.9	86.4	1k4c(A:C)	70.3	-216.1	66.7	52.2	2vdb(A:B)	77.4	-562.9	56.3	58.3	1ghq(A:B)	-0.5	-175.5	30.0	42.1
1efn(B:A)	30.0	173.4	78.8	89.7	1fc2(C:D)	23.1	-93.1	66.7	70.6	1f6m(A:C)	14.6	-307.7	56.0	63.8	3bp8(A:C)	57.9	-429.9	30.0	53.0
1buh(A:B)	70.5	151.9	78.8	71.1	2jel(H:P)	74.0	18.2	66.7	75.9	1e4k(A:C)	-41.5	-385.7	55.1	48.0	1azs(A:C)	-65.7	-331.5	28.6	51.4
2sni(E:I)	-125.0	-1.9	78.8	87.5	1zhh(A:B)	-84.0	-537.9	66.7	64.3	1zli(A:B)	-100.2	-164.6	54.1	52.4	1he8(B:A)	64.0	-323.3	27.4	40.0
1mlc(B:E)	74.4	-133.7	78.8	62.0	1gla(G:F)	-26.3	-232.4	66.7	65.4	1kxp(A:D)	189.4	-311.2	54.0	54.8	2fd6(H:U)	78.6	-317.1	27.3	31.6
1qfw(H:A)	36.5	150.4	78.6	45.4	1ml0(A:D)	-95.8	-641.1	66.7	60.4	1clv(A:I)	0.3	-648.0	54.0	79.1	1fqj(A:B)	234.8	326.9	26.4	25.9
1xd3(A:B)	-5.2	-240.3	78.0	63.4	1z0k(A:B)	9.7	-84.1	66.6	80.0	1de4(A:C)	123.1	-535.6	54.0	49.3	1syx(A:B)	116.4	113.1	26.3	66.7
2mta(L:A)	-55.7	70.5	77.4	80.0	2nz8(A:B)	52.1	36.3	66.6	77.1	1jiw(P:I)	110.3	-628.9	53.9	66.7	2b42(A:B)	103.6	-199.4	24.0	23.6
1nw9(B:A)	-120.1	-333.9	77.3	80.0	1e96(A:B)	110.7	-120.4	66.6	60.6	2b4j(A:C)	94.0	-120.6	53.8	66.7	1eaw(A:B)	12.0	-173.1	23.2	74.3
2c0l(A:B)	130.2	-225.3	76.7	78.1	2oor(A:C)	-50.4	-839.7	66.6	41.0	1ezu(C:B)	-103.2	-172.2	53.1	66.7	2pcc(A:B)	47.3	98.9	22.2	30.3
1iqd(A:C)	-14.3	-261.7	76.5	52.2	2ajf(A:E)	59.4	-194.7	64.8	57.1	1rlb(B:E)	-69.1	-322.3	52.7	63.2	1gxd(A:C)	45.2	-680.4	21.9	72.1
1nsn(L:S)	73.6	122.8	76.2	38.7	1ahw(B:C)	262.7	388.1	64.7	80.0	1ibr(A:B)	234.0	-850.1	51.3	38.5	2j7p(A:D)	208.9	122.5	21.7	30.2
1nca(H:N)	146.6	78.6	75.9	66.6	1lfd(B:A)	85.3	-28.0	64.5	85.7	2ot3(B:A)	-165.8	-494.1	51.1	52.4	1h1v(A:G)	115.0	-60.2	20.4	38.9
2z9k(A:B)	67.0	-89.9	75.6	83.3	1ay7(A:B)	123.2	-30.3	64.5	77.4	1cgi(E:I)	-186.4	-383.8	51.0	80.0	2oza(B:A)	287.3	-5.2	20.2	39.1
1grn(A:B)	189.3	-80.6	75.6	66.7	2btf(A:P)	165.6	102.3	64.0	50.0	1akj(A:D)	108.3	11.1	51.0	61.6	1kxq(H:A)	63.7	-497.6	19.7	28.1
1bgx(L:T)	127.3	-727.9	75.3	59.5	1bvk(E:F)	76.8	150.6	64.0	46.1	1f34(A:B)	-70.5	-376.9	49.3	66.6	1jwh(C:A)	-27.8	-305.7	18.7	35.3
1ppe(E:I)	-54.5	-6.3	75.0	72.8	1h9d(A:B)	12.9	167.7	63.6	72.4	1fsk(C:A)	60.7	-19.8	48.3	45.2	1bvn(P:T)	-43.9	-785.8	18.5	65.1
2cfh(A:C)	-162.0	-435.9	74.4	73.9	7cei(A:B)	216.5	192.5	63.2	68.8	1ak4(A:D)	-48.6	60.8	47.1	56.0	2o3b(A:B)	119.0	-17.4	14.3	28.6
1fcc(A:C)	247.3	160.9	74.1	66.7	1wej(L:F)	117.5	48.0	63.2	50.0	1mq8(A:B)	40.7	-56.3	46.7	84.9	1r8s(A:E)	38.2	90.0	12.6	57.7
2uuy(A:B)	-10.2	-127.7	73.5	86.9	1ib1(A:E)	163.1	240.4	62.8	63.4	2a5t(A:B)	107.0	-227.5	46.5	48.9	2hqs(A:H)	190.9	-202.6	10.5	22.6

^a^E_*act*_ is ACE score for the experimental structure on the data set.

^b^E_*pre*_ is ACE score for the prediction complex on the data set.

^c^F_*r*_ (%) is the F-score of our method for the receptor protein on the data set.

^d^F_*l*_ (%) is the F-score of our method for the ligand protein on the data set.

**Figure 5:**
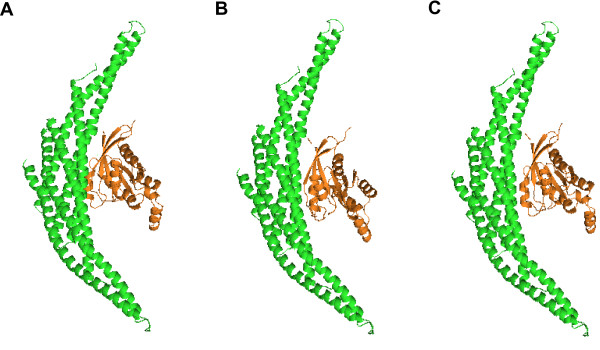
**Configuration discovered by DoBi and ZDOCK for 1i4d.** (**A**) is the configuration by DoBi; (**B**) is the configuration by ZDOCK; (**C**) is the experimental structure.

We calculate the docking results of 1i4d. The *C*_*α *_iRMSD values between the experimental structure and the configurations by DoBi and ZDOCK are 2.97Å and 1.97Å, respectively. DoBi improves F-score value of ligand protein by 2.7%, and that of receptor protein by 0.4%. The configurations produced by methods are shown in Figure 5.

### Factors affecting the performance of DoBi

We notice that DoBi performed badly on a few specific instances. We analyze this performance issue with Table 13, which compares the ACE scores for the experimental structures and predicted complexes, for the bound states of proteins in the benchmark v4.0. Among the 176 complexes, only 43 of them have an ACE score for experimental structures lower than that of the predicted complexes. This implies that in 133 cases, DoBi is able to find a configuration of a lower score than the experimental structures. These anomalies suggest that the score function currently used in DoBi may be inaccurate, and this inaccuracy may have contributed to the poorly performed cases of DoBi. We also note that the search space currently explored by our method is incomplete, and this may have contributed as well to the inaccuracy of DoBi in some cases.

**Figure 6:**
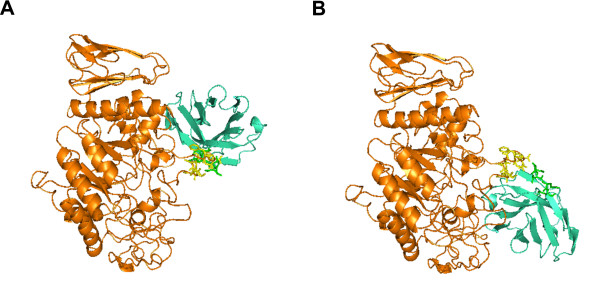
**DoBi fails to solve the instance 1kxq(H:A).** (**A**) is the predicted complex; and (**B**) is the experimental structure.

Figure 6 shows the protein complex incorrectly predicted by DoBi as well as the experimental structure for 1kxq(H:A). The iRMSD between the two complexes is 18.87Å. The ACE score of the docking structure predicted by DoBi, -497.6, is lower than the ACE score of the experimental structure, 63.7. The binding sites predicted by DoBi are incorrect as well.

## Conclusions

In this work, we proposed an approach to identify binding sites in protein complexes by docking protein subunits. The method is implemented in a program called DoBi. DoBi consistently and significantly performed better than existing techniques in predicting binding sites in experimental results.

We identify a few potential areas for future improvements to our method. The first area to work on is in the energy function used. Currently, DoBi uses a simple score function. As suggested by the experiment results, a better energy function is able to improve the performance of DoBi.

A second area for improvement is in our current assumption that protein structures are rigid when binding. In reality, protein structures may vary sightly or even dramatically when they bind. Hence, further studies on this issue are very much in demand.

Although our method shows better overall performance, there are some protein complexes where other methods outperformed DoBi. It will be beneficial if we could combine the strengths of these existing programs with DoBi, to come up with a more reliable method.

## Endnote

^a^The initial two letters from each of the two words, Docking and Binding, were taken.

## Competing interests

The authors declare that they have no competing interests.

## Authors’ contributions

FG participated in the design of the study, performed the statistical analysis, and is in charge of the software package development. SL participated in the experiment design and drafted the manuscript. LW conceived of the study, participated in its design, and helped to draft the manuscript. DZ is heavily involved in the computation of the tables. All authors read and approved the final manuscript.
